# Bioactive Compounds, Antioxidant Activity, and Sensory Analysis of Rice-Based Extruded Snacks-Like Fortified with Bean and Carob Fruit Flours

**DOI:** 10.3390/foods8090381

**Published:** 2019-09-02

**Authors:** Claudia Arribas, Blanca Cabellos, Carmen Cuadrado, Eva Guillamón, Mercedes M. Pedrosa

**Affiliations:** 1 Food Technology Department, SGIT-INIA, Ctra de La Coruña, Km 7.5., 28040 Madrid, Spain; 2 Centre for the Food Quality, INIA, C/Universidad s/n, 42004 Soria, Spain

**Keywords:** legumes enrichment, galactosides, phytate, protease inhibitors, phenols

## Abstract

Generally, extruded gluten-free foods are mostly phytochemically deficient. In this study inositol phosphates, α-galactosides, lectins, protease inhibitors, and phenols, their antioxidant activity and sensorial analysis of some rice/bean/whole carob fruit flour blends were determined in unprocessed (controls) and extruded formulations. The fortification of rice-based extrudates with both legumes has a positive influence on both their bioactive compound content and their acceptability by consumers. The extruded formulations contained around twice as much (*p* < 0.05) total α-galactosides than their unprocessed counterparts. Extrusion significantly reduced the phytic acid content (10%) and significantly increased the less phosphorylated forms (16%–70%). After extrusion, the lectins and protease inhibitors were eliminated. The different phenolic compounds mostly increased (11%–36%), notably in the formulations with carob fruit. The antioxidant activity and the different groups of phenols showed a positive correlation in the extrudates. All the experimental extrudates had higher amounts of bioactive compounds than the commercial extruded rice. Considering the amount of phytochemicals determined in the novel gluten-free extrudates and the scores of sensorial analysis, formulations containing 20%–40% bean and 5% carob fruit could be adequate in promoting health-related functions, helping to increase pulse consumption, and allowing the food industry to satisfy consumers’ requirement for functional foods.

## 1. Introduction

Nowadays, consumers demand ‘free-from’ foods to improve their health and wellbeing. Among the free-from foods, gluten-free (GF) products have become very popular, mainly because consumers perceive these GF foods as healthier than the corresponding gluten-containing counterparts. However, it has been reported that a GF diet might not be recommended for the general population due to the nutritional deficiencies associated with GF diets [1]. 

There are different, naturally whole GF grains that have shown to contain good nutritional quality and good bioactive compound content, which are intrinsically related to healthy benefits. Dehusked rice (*Oryza sativa* L.) is reckoned as the most appropriate ingredient for the formulation of a wide variety of GF foods, such as breakfast cereals, cereal-based snacks, or dietetic foods. However, although whole rice can supply many bioactive compounds, after dehusking and polishing, the obtained white rice loses many phytochemicals, such as phytate, polyphenols, and protease inhibitors [2]. Beans, like other pulses, are well-known sources of phytochemicals (e.g., α-galactosides, phytates, protease inhibitors, phenols, or lectins), which are traditionally considered as antinutritional factors since they can reduce the digestion/absorption of some nutritive compounds or cause intestinal discomfort. Nevertheless, nowadays, some of these compounds are recognised as bioactive compounds that provide beneficial health effects (prebiotic, anti-tumour, or anti-inflammatory effects), playing a pivotal role in reducing the risk or the prevention of heart diseases, some cancers, obesity, type 2 diabetes, and Parkinson’s disease [3,4,5,6]. However, their antinutrient or pro-nutrient effect has been related to the amount present in foods, as well as to interactions with other compounds present in the diet [4,5]. The fruits of the carob tree (*Ceratonia siliqua* L.), an underutilised leguminous tree from the Mediterranean region, have been traditionally exploited as a feed ingredient. The carob seeds are mostly utilised for the production of locus bean gum or carob bean gum (E-410), which is of great importance for the food industry as a stabiliser or thickener. The seedless pods (named kibble) have mainly been utilised for feed and as a cocoa substitute in cakes, breads, confectionery, or drinks because of its high content of dietary fibre and soluble sugars [7]. Furthermore, carob fruits contain a range of phytochemicals, mainly polyphenols and dietary fibre, as well as low amounts of α-galactosides and inositol phosphates [7,8].

Recent evidence suggests that a GF diet reduced beneficial gut bacteria as a result of the reduced intake of oligosaccharides from wheat (such as oligofructose and inulin) with a prebiotic action [1], therefore, the use of legumes, such as bean and carob fruit, would be of great interest as functional ingredients to formulate pro-health or functional foods, providing bioactive compounds to white-rice based products, and increasing the protein and dietary fibre content in these foods [2]. Moreover, the new food products fortified with legumes would lead to increasing pulse consumption and promote a healthy Mediterranean diet.

Extrusion is a versatile technique used by the industry that enables the formulation of different types of ready-to-eat products such as snacks and breakfast cereals with a high rate of consumption, mainly by young people. They are mainly based in rice or corn flours and/or starches and tend to have low nutritional quality and high calorie content [9,10]. A recommended solution to enhance their nutritional quality is the enrichment of rice flour with legume flours [2]. Different pulses (beans, peas, lentils, chickpeas, soy, etc.) have been treated by extrusion/cooking, though they are generally blended with rice or corn starch to develop good expanded products [11,12,13,14]. It has been reported that extrusion is an efficient process to modify the content of some phytochemicals, although with ambivalent results, since the extrusion conditions as well as the raw materials used can increase or reduce the amount of these compounds [3,11,13,15,16], therefore, it is necessary to determine these changes to obtain maximum health benefits in novel extrudates. 

Although Ravindran, et al. [17] described the use of carob bean gum to enhance the characteristics of extruded rice-pea formulas, to the best of our knowledge, there is little information [3,10] about the utilisation of carob fruit flours in the preparation of extruded foods. Furthermore, according to the literature consulted, there is no information on the bioactive compound composition of novel GF extrudates based on the linked use of rice, whole carob fruit, and bean flours. 

Therefore, the main aim of this research was to study the extrusion effect on some phytochemicals content (α-galactosides, inositol phosphates, lectins, protease inhibitors, and phenolic compounds), as well as the antioxidant activity of various rice-based blends enriched with bean and whole carob fruit flours. The data obtained from the extrudates was compared with their unprocessed equivalents (controls) and with a commercial extruded rice. The results will update the knowledge related to the presence of bioactive compounds in innovative GF products made from legume formulations. 

## 2. Materials and Methods

### 2.1. Raw Materials and Formulated Flours

The ingredients used for the formulation of the extrudates were white rice (*O. sativa* var. Montsianell), bean (*Phaseolus vulgaris* var. Almonga), and whole carob fruit (*Ceratonia siliqua* var Negreta and Roja). The ingredients were obtained from Cámara Arrocera de Amposta (Tarragona, Spain), a local farmer (Benjamín Rodríguez Álvarez, León, Spain), and Armengol Hermanos (Tarragona, Spain), respectively. The seeds and fruits were powdered using a Retsch SK1 mill (Haan, Germany) fitted with a 1 mm sieve. Different percentages of rice (50%–80%), bean (20% or 40%), and whole carob fruit (5% or 10%) flours were mixed to produce six formulations coded as 20.0, 20.5, 20.10, 40.0, 40.5, and 40.10. The codes used correspond to the following: The first number corresponds to the bean percentage and the second one to the carob fruit percentage included in the blends. The results from the extrudates were compared to an extruded rice sample (100%) obtained in the market. 

### 2.2. Extrusion Process

A Clextral EVOLUM 25 twin-screw extruder (Clextral, Firminy, France) from CARTIF (Valladolid, Spain) was utilised. The extruder was equipped with a 25 mm screw diameter and a 600 mm barrel length. The mixtures were added to the extruder at a rate of 25 kg/h. The last barrel temperature was 125 °C. The screw speed was 900 rpm for 20% bean formulations and 950 rpm for 40% bean formulations. The rate of water addition was 2.5, 3.0, and 3.2 kg/h for samples without carob fruit flour, with 5% carob fruit flour, and with 10% carob fruit flour, respectively. The extrudates were reduced to flour using a Tecator mill (Cyclotec 1093, Höganäs, Sweden) equipped with a 1 mm screen and stored at room temperature in polyethylene bags until the analysis was performed. 

### 2.3. Analysis of Bioactive Compounds

The analysis of raw materials, unprocessed mixtures (as controls), and extruded samples, as well as the commercial extruded sample was performed. 

#### 2.3.1. Soluble Sugars and α-Galactosides

Sugars were extracted and then analysed with a HPLC system coupled to a refractive index detector (Beckman System Gold Instrument, Los Angeles, CA, USA) following the method described by Pedrosa, et al. [18]. A total of 20 μL of each sample was injected in a spherisorb-5-NH2 column (250 × 4.6 mm i.d., Waters, Milford, MA, USA) equilibrated with acetonitrile/water (60:40, *v*/*v*) at a flow rate of 1 mL/min. Each individual sugar was identified and quantified by using the corresponding calibration curves from external standards (Sigma, St. Louis, MO, USA). Ciceritol was obtained from Dr. A. I. Piotrowicz-Cieslak (Olsztyn-Kortowo, Poland). A linear response was observed in the range (0–4 mg/mL), with a correlation coefficient of 0.99. The curves obtained were: Sucrose (*y* = 0.193x − 0.001), galactinol (*y* = 0.227x − 0.014), raffinose (*y* = 0.193x + 0.003), ciceritol (*y* = 0.295x + 0.030), and stachyose (*y* = 0.217x − 0.008). Original HPLC chromatograms of sugars and galactosides of all the analysed samples are presented in supplementary Figure S3.

#### 2.3.2. Inositol Phosphates

Individual inositol phosphates (IP3–IP6) were extracted and determined by HPLC and refractive index detection according to Burbano, et al. [19]. A total of 10 μL of Aliquots were injected on a PRP-1 column (150 × 4.1 mm i.d., 5 μm, Hamilton, Reno, Nevada, USA), and was maintained at 45 °C. The mobile phase was a mixture of methanol/water (52/48, *v*/*v*) with 0.8% of tetrabutyl ammonium hydroxide (40% in water, Sigma, St. Louis, MO, USA), 0.05% of 91% formic acid (Sigma, St. Louis, MO, USA), 100 μL of a phytic acid hydrolysate (6 mg/mL), and the pH was adjusted to 4.3 with 5 M of sulphuric acid. The flow rate was 1 mL/min. The quantification was developed by using a calibration curve obtained from sodium phytate (0–5 mg/g, *y* = 0.144x + 0.016, *R*^2^ = 0.99) (Sigma, St. Louis, MO, USA). Original HPLC chromatograms of IP3-IP6 of all the analysed samples are presented in supplementary Figure S4.

#### 2.3.3. Protease Inhibitors

The trypsin inhibitors (TI) and chymotrypsin inhibitors (CI) were obtained following the method described by Pedrosa, et al. [20]. The trypsin inhibitor units (TIU) per mg of flour were determined as described by Welham and Domoney [21]. One TIU was defined as that which gave a reduction in absorbance units measured at 410 nm of 0.01 relative to the trypsin control reaction in a 10 mL assay volume. Chymotrypsin inhibitor units (CIU) per mg of flour were determined according to Sathe and Salunkhe [22]. One CIU was defined as an increase of 0.01 absorbance units at 256 nm of the reaction mixture relative to the chymotrypsin control reaction.

#### 2.3.4. Lectins

The lectin content in the raw materials was determined using a haemagglutination assay with trypsin-treated rat blood cells (Wistar rats, Animal-housing unit of Complutense University, Madrid, Spain). To quantify the *P. vulgaris* lectin (PHA) in raw bean- and bean-containing formulations, a competitive indirect ELISA assay was carried out according to Pedrosa, Cuadrado, Burbano, Muzquiz, Cabellos, Olmedilla-Alonso, and Asensio-Vegas [20]. One haemagglutination unit (HU) was defined as the amount of material (mg) causing 50% agglutinated erythrocytes. The values were expressed as HU/kg flour for the haemagglutination assay, and as a % PHA for the ELISA assay. In each ELISA assay, two bean cultivars (Procesor and Pinto) were included as positive and negative controls, respectively. The calibration curve of a standard PHA (0–500 ng/mL) used for quantification of bean lectins was *y* = −0.4311Ln(x) + 2.711 (*R*^2^ = 0.98).

#### 2.3.5. Phenolic Compounds

Phenolic compounds were extracted twice using a mixture of methanol-HCl (1000/1)/water (80:20, *v*/*v*) at room temperature for 16 h [23]. A total of 1 mL of the combined extracts was mixed with 1.5 mL of a solution of 2% HCl in ethanol/water (80/20, *v*/*v*). These solutions were utilised for the quantification of anthocyanins, flavonoles, tartaric esters, and total phenols by a spectrophotometric method (Beckman DU-640, Los Angeles, CA, USA) that monitored the absorbance at 520, 360, 320, and 280 nm, respectively [24]. The extracts were also utilised for the determination of the antioxidant activity by using the ORAC method [25]. Calibration curves obtained from commercial cyanidin-3-glucoside (C3Glc) (0–20 μg/mL; *y* = 0.060x + 0.060; *R*^2^ = 0.99) (Extrasynthèse, Germay, France), quercetin (Q) (0–80 μg/mL; *y* = 0.059x + 0.083; *R*^2^ = 0.99), caffeic acid (CA) (0–30 μg/mL *y* = 0.095x + 0.009; *R*^2^ = 0.99), and catechin (C) (0–200 μg/mL *y* = 0.012x + 0.005; *R*^2^ = 0.99) (Aldrich, Munich, Germany) were utilised to quantify anthocyanins, flavonols, tartaric esters, and total phenols, respectively, according to Oomah, et al. [24].

### 2.4. Sensory Evaluation

The 6 extrudates (Ex-20.0, Ex-20.5, Ex-20.10, Ex-40.0, Ex-40.5, and Ex-40.10) were evaluated by 10 semi-trained panellists. Samples were codified with random numbers, placed in small plastic boxes, and given to the panellists. They were instructed to cleanse their mouth with water between each sample evaluated and they were provided with written instructions. Extrudates were evaluated for colour, odour (intensity, roasted, cooked pulse, nuts, unpleasant odours), flavour (intensity, salty, pulses, unpleasant flavours), texture (crunchiness, hardness, adhesivity, fracture, chewiness), and overall quality, according to a nine-point scale where 9 = extremely like or high intensity and 1 = extremely dislike or low intensity). 

### 2.5. Statistical Analysis

A representative and homogeneous amount (25 kg) of each formulation was split into in two parts and then extruded. The chemical analyses of raw materials, unprocessed, and extruded samples and the commercial sample were carried out in quadruplicate. Data are reported as mean ± standard error. A prior analysis of the normality and homogeneity of variance of all variables was performed using Shapiro–Wilks and Levenes’s test, respectively. One-way ANOVA and a Duncan’s multiple range test were used to analyse data at the 95% confidence level (*p* < 0.05). Pearson’s correlation coefficients between the different bioactive compounds and the ORAC values were determined. Moreover, a principal component analysis (PCA) of the standardised data of the measured variables was performed to ensure their reliability and to obtain meaningful interpretations of the results (Supplementary Materials). All the statistical analyses were developed using Statgraphics Centurion XVII.II software (Graphics Software System, Rockville, MD, USA).

## 3. Results and Discussion

### 3.1. Effect of Bean and Whole Carob Flour Fortification on the Content of Some Bioactive Compounds of Rice-Based Unprocessed Formulations

As observed in Table 1, Table 2, Table 3 and Table 4, raw bean flour showed the highest content of α-galactosides (raffinose and stachyose), inositol phosphates (IP3–IP6), and protease inhibitors. Raw carob fruit flour contained the lowest amount of total inositol phosphates but also the highest sucrose, total phenols, and antioxidant activity. Regarding the haemagglutination activity measured using trypsin-treated rat blood cells (data not shown), raw bean flour showed the highest lectin content (10 HU/kg), followed by carob fruit (0.32 HU/kg), and rice (0.16 HU/kg). Moreover, raw rice contained low amounts of sucrose and TI. The bioactive compound content established in the raw materials was close to that reported in the literature [3,4,25,26].

To study the extrusion effect on the phytochemical content of the novel formulations, the unprocessed (NE-) formulations were used as controls. The rice/legume blends are a complex matrix that can affect the extraction of bioactive compounds [12,18,20], therefore, it is necessary to know the amount of different phytochemicals in the unprocessed samples.

The addition of whole carob fruit and bean flour had a positive impact on the phytochemical composition of non-extruded (NE-) mixtures. The higher legume percentage produced more α-galactosides (Table 1) and inositol phosphates (Table 2) content in the NE-blends, with stachyose and phytic acid (IP6) being the main compounds detected. The amount of sugars in the NE-blends showed, in general, the following pattern: Sucrose > stachyose > raffinose > galactinol > ciceritol. The total amount of galactosides, on average, was 11.27 mg/g in the blends with 20% bean and 16.43 mg/g in the blends with 40% bean. A considerable increase in sucrose (Table 1) and phenolic compounds (Table 4) content was detected in the NE-samples containing carob fruit flour (NE-20.5, NE-20.10, NE-40.5, and NE-40.10) due to the high content of both compounds determined in raw whole carob fruit. In relation to the protease (trypsin and chymotrypsin) inhibitor content (Table 3), the lowest activities corresponded to the formulations with 20% bean and the highest values coincided with the 40% blends. The addition of carob fruit flour did not produce significant changes (*p* > 0.05) in the protease inhibitor activities analysed. The content of lectin (PHA) in the different NE-formulations, evaluated by competitive ELISA did not show significant (*p* > 0.05) differences, ranging from 0.035% to 0.108% PHA. As carob fruit contained higher amounts of phenols than bean and rice, the levels of the different phenolic compounds in NE-blends increased as a function of increasing the proportion of carob. The same tendency was found for the ORAC. A positive correlation was observed among total phenols and ORAC (*R^2^* = 0.58, *p* < 0.05) and flavonols and ORAC (*R^2^* = 0.59, *p* < 0.05). Moreover, the incorporation of carob fruit and bean in the formulation of different rice-based blends had a positive impact on the bioactive compound content. This was also observed by other authors who added different legumes (navy bean, kidney bean, small red bean, pea, faba bean, or chickpea) to unprocessed formulations based on starch or flour from corn or rice [2,11,14,25,27].

### 3.2. Extrusion Cooking Impact on the Bioactive Compounds of Rice-Based Formulations

#### 3.2.1. Soluble Sugars and α-Galactosides

The amount of soluble sugars and α-galactosides in the different experimental extrudates and the commercial extruded rice is presented in Table 1. Similar to the unprocessed (NE-) blends, the main sugars found in all the extrudates were sucrose, raffinose, and stachyose. In general, the sugar content pattern in the extrudates was the same as in the NE-counterparts. Extrusion produced an increase (*p* < 0.05) in all the analysed sugars. Regarding the total α-galactosides content, the extrudates with 20% (Ex-20) and 40% (Ex-40) bean presented, on average, 1.78 and 2.03-folds more α-galactosides than their equivalent NE-counterpart (NE-20 and NE-40), respectively. This increase could mostly be attributed to the release of carbohydrates linked to other macromolecules as well as to a mechanical alteration of the structure of the food matrix during extrusion, including cell wall damage, which increased the porosity, thus enhancing the diffusion of the extracting solvent within the food matrix, and subsequently improving the sugar extraction [12,18]. These results were similar to those described for extruded pea and lentil flours [3,12]. However, a reduction in the sugar content of extruded legumes has also been reported [28,29,30] although these authors reported that the extrusion effect on the galactosides content depends on the moisture and temperature of the extrusion process, as well as the seed and/or the formulation used in the development of the extruded products.

The commercial sample did not contain α-galactosides. Compared to the commercial sample, extrudates without carob fruit flour contained between four and seven times less sucrose, while the extrudates containing 5%–10% carob flour showed, on average, between 1.72 (Ex-40.10) and 3.23 (Ex-20.5) times more sucrose because of the high sucrose amount provided by this raw material. 

From a physiological perspective, sugars of the raffinose family are recognised as accounting for flatulence associated with legume consumption. However, different studies have reported that these galactosides can be considered as prebiotics [4,5,31]. Their presence in the diet has been associated with prebiotic activity, increasing the bifidobacterias population in the human colon, stimulating the immune system, and reducing diarrhoea or constipation. Moreover, the colonic flora can ferment these sugars, producing a mixture of short fatty acid that reduces glycaemic symptoms and cholesterol once assimilated into the intestine [5,6]. Therefore, the increase in the total amount of α-galactosides after extrusion can be considered as an added-value attribute of the extrudates and allows bean and carob fruit be taken into account as an added-value component in the extruded formulations. 

#### 3.2.2. Inositol Phosphates

It is known that inositol hexakisphosphate, phytate, or phytic acid (IP6) form complexes with some minerals (iron, zinc, or calcium), which negatively affects their absorption. However, the lower phosphorylated forms (IP–IP4) are considered to have a significant function in human health, boosting the assimilation of minerals, preventing the formation of kidney stones, and performing key roles in some disorders, such as type 2 diabetes, some types of cancer, and irritable bowel syndrome [4,5,6,32].

As observed in the NE-blends, IP6 was the principal inositol phosphate detected in all the extrudates (Table 2). Extrusion cooking reduced (*p* < 0.05), on average, 10% of the IP6 content, leading to less phosphorylated forms [3,12,32]. After extrusion, IP4 and IP5 showed a significant increase (16%–52% and 30%–70%, respectively) compared to their corresponding NE-equivalents. A reduction (*p* < 0.05) in the total IP content was also found (7%–15%) for the Ex-20 samples compared to their respective unprocessed formulations, mostly due to the IP6 reduction. These results were in accordance with those described for extruded faba bean, pea, kidney bean, and chickpea [11,25,29,30]. Although IP6 was reduced (5%–10%) in the Ex-40 samples, the total IP content of these samples showed a slight increase (3%–4%), which did not differ significantly from their NE-40 blends, similar to the results of Lombardi-Boccia, et al. [33] for extruded bean, faba bean, chickpea, and lentil. The extent of the changes on the inositol phosphate content is dependent on the raw materials and/or the composition of the formulations, as well as the extrusion conditions used in the formulation of the extrudates [14,30,32]. Regarding these results, extrusion might be considered a good processing technology to reduce IP6 content by retaining the advantageous lower phosphorylated forms in the innovative extrudates. 

The commercial extruded rice contained 1.6 to 2.7 times less total IP and 3–5 times less IP6 than the Ex-formulations. This could be due to inositol phosphates (in the experimental extrudates) that were mainly provided by the bean flour.

#### 3.2.3. Protease Inhibitors and Lectins

As shown in Table 3, the extrusion process eliminated TI and CI activities, as well as lectin content in all the extruded blends, which corroborated the heat-sensitive nature of both phytochemicals. Similarly, several authors found a reduction of TI and lectins by 100% in extruded corn/bean, pea, chickpea, kidney bean, and faba bean [11,12,25,27,34]. The commercial extruded rice showed a small TI activity (0.09 TIU/mg), while CI activity and lectins were not detected. It has been reported that protease inhibitors and lectins are key parameters in establishing food quality [4,26,32], since they hamper protein digestion and the assimilation of nutrients, respectively. Therefore, the absence of both compounds in all the extrudates may improve their nutritional quality [25,26,32]. Moreover, as PHA is considered a toxic compound (causing vomiting, bloating, or diarrhoea in humans) [32], from the perspective of lectin toxicity, these results demonstrate that all the extrudates are safe.

#### 3.2.4. Phenolic Compounds and Antioxidant Activity

Phenolic compounds are considered to be natural antioxidants that can extend the shelf-life of food products [30,35]. The phenolic compound (anthocyanins, flavonols, tartaric esters, and total phenols) content and the antioxidant activity (ORAC) of the extrudates is shown in Table 4. The extrusion process affected the studied phenolic groups to a different extent. In general, the Ex-formulation showed a significant increase in the tartaric esters (on average 11%), anthocyanins (24%), and total phenols (36%) content compared to their NE-counterpart, while the flavonols content in the extrudates did not vary significantly from their NE-formulations. The observed increase could be related to the lack of effluents during extrusion, unlike traditional processing (soaking and/or cooking or autoclaving) that prevent the leaching of water-soluble phenols [20,30]. As expected, the highest increases in total phenols (30%–56%) corresponded to the extrudates that included carob bean flour in their composition (Ex-20.5, Ex-20.10, Ex-40.5, and Ex-40.10). The carob fruit pods are high in phenolic compounds bound to the dietary fibres and the extrusion could lead to the release of some non-available phenols bound to the cell wall, thus growing their extractability [3,15].

Regarding the ORAC, the greatest values were found in the extrudates containing 40% bean and 5%–10% carob fruit. The majority of the extruded formulations showed a slight increase (on average 5.4%) in ORAC, albeit, in general, the differences between the ORAC values of each corresponding pair of NE/Ex-formulations were not significant (*p* > 0.05). A positive correlation was determined among anthocyanins, tartaric esters, and ORAC (*R^2^* = 0.63 and 0.58, *p* < 0.05, respectively). However, the antioxidant activity is conditioned by the type of compound and its amount in the sample [24]. The increase found in ORAC values after extrusion might also be attributable to the existence of other food components (e.g., proteins or aromatic compounds) or to the creation of Maillard reaction products throughout the extrusion that contribute to the antioxidant activity analysed [3,11,27]. 

As reported by different authors [11,14,15,34], extrusion can produce rises or decreases in both the phenol content and the ORAC of many pulses, and they concluded that extrusion at moderately low moisture (<14%) and temperatures below 120 °C retained higher amounts of phenols and showed greater antioxidant activity. 

Compared to the commercial sample, the Ex-20.10, Ex-40.5, and Ex-40.10 formulations presented a higher (*p* < 0.05) amount of total phenols (approximately 1.5 times) and anthocyanins (around 1.6 times), as well as a higher ORAC value (from 1.2 times to 1.4 times).

Various studies reported health benefits associated with different phenols and their antioxidant capacity, such as a lower risk for inflammatory processes or colon cancer [16,23,35]. Therefore, considering the results for the phenolic compounds content and antioxidant capacity, the fortification of rice-based extrudates with both whole carob fruit and bean would be helpful in developing healthy foods. 

A PCA was performed to classify and characterise the Ex- and NE- formulations according to their phytochemical content. The PCA on the standardised phytochemical data of the NE- and Ex- samples showed that three principal components explained 86.85% of the total variance (supplementary file, Figures S1 and S2, Tables S1 and S2). Total galactosides and the different phenolic compounds had more weight in the characterisation of the samples by PC1, whereas the thermo-labile compounds (lectins and protease inhibitors) had more weight in the characterisation of the samples by PC2. The variance of PC3 was mainly explained by the ORAC.

### 3.3. Sensory Evaluation of the Extrudates

**Figure 1:**
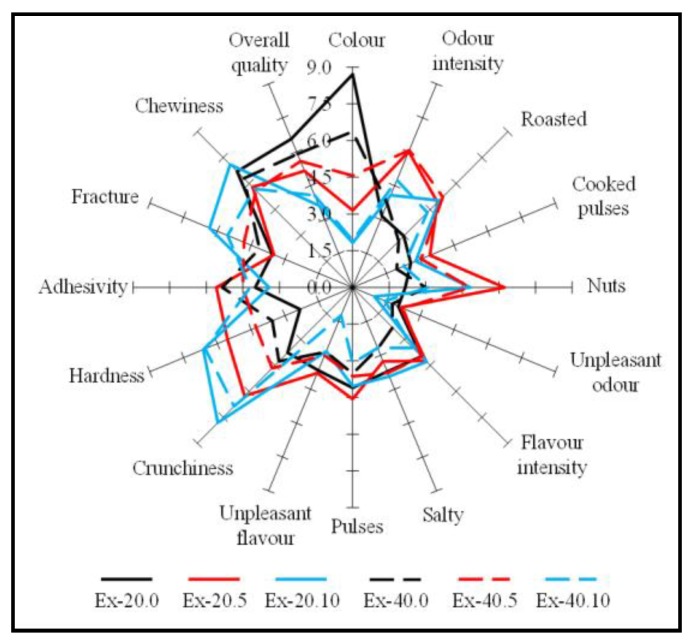
Sensory analysis of the six different extrudates formulated with different proportions of rice, bean, and carob fruit flours. Sample code: Ex-20.0 (20% bean; 0% whole carob fruit); Ex-20.5 (20% bean; 5% whole carob fruit); Ex-20.10 (20% bean; 10% whole carob fruit); Ex-40.0 (40% bean; 0% whole carob fruit); Ex-40.5 (40% bean; 5% whole carob fruit); and Ex-40.10 (40% bean; 10% whole carob fruit).

In the sensory analysis, the panellists determined how each extrudate compared with the other by considering a number of different attributes related to colour, odour, flavour, and texture. The results of the sensory analysis are shown in Figure 1. The higher scores represent better acceptance of the attribute evaluated. 

Textural attributes, mainly crunchiness and hardness, are largely determined to attest to the quality and the consumer acceptability of extruded products, such as snacks or breakfast cereals. The extrudates including carob fruit in their composition showed better textural scores with high crunchiness and low adhesiveness. Crunchiness results were positively correlated (*R^2^* = 0.55, *p* < 0.001) with the presence of carob flour in the formulation. 

The colour scores were affected (*p* < 0.05) by the presence of carob flour. A higher amount of carob flour caused a lower preferred colour by the panellists. A strong negative correlation between colour and the presence of carob flour (*R^2^* = −0.77, *p* = 0.001) was found. This could be related to the brown colour of the products formulated with carob flour, leading to a slight rejection of these products. The analysis of the scores of the different aroma and taste parameters tested did not show any correlation with the amount of legumes in the extrudates and no unpleasant odours or flavours were detected. 

Regarding the overall quality results, the amount of bean (20% or 40%) in the formulations did not affect (*p* > 0.05) the overall quality of the extrudates. The extrudates containing carob flour showed lower scores, however, the differences between them were not significant (*p* > 0.05). The extrudates elaborated only with rice and bean showed the highest scores, although the addition of 5% carob fruit flour did not significantly affect the overall quality of these extrudates, and therefore these novel extrudates would be well accepted by consumers. 

## 4. Conclusions

From the results obtained, it can be seen that the fortification of rice-based GF puffed products with carob fruit and bean flours had a positive impact on their bioactive compound content. The fortification with carob fruit flour improved their textural attributes and did not significantly affect their overall quality.

The extrusion process affected the studied phytochemicals to a different extent. While total α-galactosides and phenols increased, the IP6 was reduced, and the lectins and protease inhibitors were eliminated. The content of bioactive compounds present in these extrudates might be enough to promote health-associated functions. Moreover, the absence of lectins and protease inhibitors enhanced the nutritional quality of the extrudates. Compared to the commercial extruded rice, all the experimental extrudates showed a higher content of bioactive compounds. Therefore, the formulation of these products would be of interest to both health-conscious consumers and the food industry, allowing it to meet consumers’ requirement for functional foods with acceptable sensory attributes.

## Figures and Tables

**Table 1:** Effect of extrusion treatment on the soluble sugars, ciceritol, and α-galactosides (mg/g dry weight) content of raw materials, the non-extruded (NE-) and the extruded (Ex-) flour formulations, and the commercial sample.

Sample	Sucrose	Galactinol	Raffinose	Ciceritol	Stachyose	Total α-Galactosides
**Bean**	30.00 ± 0.95 ^e,f,g^	2.29 ± 0.05 ^a,b^	5.92 ± 0.09 ^c^	0.34 ± 0.01 ^a^	26.85 ± 0.25 ^g^	32.77± 0.25 ^g^
**Carob fruit**	150.46 ± 10.04 ^h^	n.d. *	5.84 ± 0.02 ^c^	n.d.	n.d.	5.84 ± 0.02 ^a^
**Rice**	2.98 ± 0.15 ^a^	n.d.	n.d.	n.d.	n.d.	n.d.
NE-20.0 **	8.65 ± 0.15 ^a,b,A^	2.53 ± 0.10 ^a,b,c,A^	2.84 ± 0.13 ^a,A^	0.78 ± 0.04 ^b,A^	8.55 ± 0.40 ^a,A^	11.39 ± 0.55 ^b,A^
**NE-20.5**	16.85 ± 0.29 ^c,d,A^	2.97 ± 0.06 ^b,c,d,e,A^	3.08 ± 0.03 ^a,A^	0.90 ± 0.02 ^b,c,A^	7.59 ± 0.17 ^a,A^	10.67 ± 0.15 ^b,A^
**NE-20.10**	29.91 ± 0.96 ^g,h,A^	3.30 ± 0.07 ^c,d,e,A^	3.14 ± 0.04 ^a,A^	0.94 ± 0.03 ^b,c,d,A^	8.61 ± 0.40 ^a,A^	11.76 ± 0.45 ^b,A^
**NE-40.0**	12.44 ± 0.15 ^c,A^	3.58 ± 0.03 ^d,e,A^	3.87 ± 0.04 ^b,A^	1.14 ± 0.05 ^c,d,e,A^	11.82 ± 0.23 ^c,A^	15.69 ± 0.25 ^c,A^
**NE-40.5**	22.83 ± 0.73 ^d,e,f,A^	3.11 ± 0.13 ^b,c,d,e,A^	3.97 ± 0.08 ^b,A^	1.19 ± 0.05 ^d,e,A^	13.37 ± 0.40 ^d,A^	17.34 ± 0.49 ^d,A^
**NE-40.10**	30.06 ± 0.41 ^h,A^	1.87 ± 0.09 ^a,A^	3.89 ± 0.09 ^b,A^	1.25 ± 0.04 ^e,A^	12.37 ± 0.28 ^c,d,A^	16.26 ± 0.48 ^c,d,A^
**Ex-20.0**	10.27 ± 0.08 ^a,b,A^	2.84 ± 0.11 ^b,c,d,A^	9.46 ± 0.35 ^e,B^	5.44 ± 0.07 ^f,B^	10.30 ± 0.43 ^b,B^	19.76 ± 0.78 ^e,B^
**Ex-20.5**	22.10 ± 0.19 ^d,e,B^	10.27 ± 0.34 ^h,B^	7.60 ± 0.27 ^d,B^	5.64 ± 0.15 ^f,B^	11.51 ± 0.50 ^c,B^	19.10 ± 0.81 ^e,B^
**Ex-20.10**	34.62 ± 0.10 ^e,f,g,A^	9.71 ± 0.44 ^h,B^	8.12 ± 0.26 ^d,B^	5.42 ± 0.07 ^f,B^	13.36 ± 0.07 ^d,B^	21.48 ± 0.33 ^f,B^
**Ex-40.0**	17.84 ± 0.16 ^c,d,A^	3.81 ± 0.10 ^e,A^	12.21 ± 0.24 ^f,B^	8.92 ± 0.07 ^h,B^	20.91 ± 0.13 ^e,B^	33.13 ± 0.50 ^g,h,B^
**Ex-40.5**	31.64 ± 0.05 ^f,g,A^	5.31 ± 0.25 ^f,B^	12.16 ± 0.15 ^f,B^	8.93 ± 0.13 ^h,B^	22.14 ± 0.20 ^f,B^	34.30 ± 0.28 ^h,B^
**Ex-40.10**	41.60 ± 0.07 ^e,f,g,B^	7.31 ± 0.33 ^g,B^	12.05 ± 0.34 ^f,B^	8.41 ± 0.01 ^g,B^	20.98 ± 0.20 ^e,f,B^	33.03 ± 0.14 ^g,h,B^
**Commercial extruded rice**	71.40 ± 1.31 ^g^	n.d.	n.d.	n.d.	n.d.	n.d.
***p* value**	<0.0001	<0.001	<0.0001	<0.001	<0.0001	<0.001

* n.d. not detected. Values are mean ± standard error (*n* = 4); mean values in the same column followed by a different superscript are significantly (*p* < 0.05) different; small superscript letters mean differences between all the samples analysed, whereas capital superscript letters mean differences due to extrusion treatment for the same formulation. ** Sample codes: 20.0 (20% bean; 0% whole carob fruit); 20.5 (20% bean; 5% whole carob fruit); 20.10 (20% bean; 10% whole carob fruit); 40.0 (40% bean; 0% whole carob fruit); 40.5 (40% bean; 5% whole carob fruit); and 40.10 (40% bean; 10% whole carob fruit).

**Table 2:** Effect of extrusion treatment on the inositol phosphate content (mg/g dry weight) of raw materials, the non-extruded (NE-) and the extruded (Ex-) flour formulations, and the commercial sample.

Sample	IP3	IP4	IP5	IP6	Total Inositol Phosphates
**Bean**	0.26 ± 0.01 ^e^	0.42 ± 0.01 ^e^	1.39 ± 0.03 ^g^	10.12 ± 0.03 ^j^	12.20 ± 0.04 ^h^
**Carob**	n.d. *	0.15 ± 0.01 ^b^	0.36 ± 0.04 ^b^	0.15 ± 0.01 ^a^	0.66 ± 0.03 ^a^
**Rice**	0.10 ± 0.01 ^b,c^	0.03 ± 0.03 ^a^	0.22 ± 0.01 ^a^	1.53 ± 0.05 ^b^	1.88 ± 0.03 ^b^
NE-20.0 **	0.22 ± 0.01 ^d,e,A^	0.24 ± 0.01 ^c,A^	0.53 ± 0.02 ^c,A^	3.32 ± 0.11 ^e,f,A^	4.31 ± 0.12 ^f,g,A^
**NE-20.5**	0.22 ± 0.01 ^d,e^	0.25 ± 0.01 ^c,A^	0.53 ± 0.01 ^c,A^	3.28 ± 0.12 ^e,A^	4.27 ± 0.10 ^f,A^
**NE-20.10**	0.22 ± 0.01 ^d,e^	0.27 ± 0.01 ^c,A^	0.57 ± 0.01 ^c,A^	3.88 ± 0.01 ^h,i,A^	4.93 ± 0.02 ^h,A^
**NE-40.0**	0.22 ± 0.01 ^d,e,A^	0.27 ± 0.01 ^c,A^	0.71 ± 0.01 ^d,A^	4.08 ± 0.06 ^i,j,A^	5.28 ± 0.07 ^i,A^
**NE-40.5**	0.22 ± 0.01 ^d,e,A^	0.27 ± 0.01 ^c,A^	0.75 ± 0.01 ^d,A^	4.18 ± 0.09 ^h,A^	5.42 ± 0.10 ^i,A^
**NE-40.10**	0.22 ± 0.01 ^d,e,A^	0.24 ± 0.01 ^c,A^	0.90 ± 0.03 ^e,A^	4.54 ± 0.04 ^i,A^	5.90 ± 0.07 ^j,A^
**Ex-20.0**	0.05 ± 0.01^a,b,B^	0.29 ± 0.01 ^c,A^	0.69 ± 0.02 ^d,B^	2.61 ± 0.03 ^c,B^	3.65 ± 0.09 ^d,B^
**Ex-20.5**	n.d.	0.29 ± 0.01 ^c,A^	0.71 ± 0.02 ^d,B^	2.98 ± 0.11 ^d,B^	3.98 ± 0.14 ^e,B^
**Ex-20.10**	n.d.	0.27 ± 0.01 ^c,A^	0.74 ± 0.01 ^d,B^	3.56 ± 0.12 ^f,g,B^	4.56 ± 0.16 ^g,B^
**Ex-40.0**	0.05 ± 0.01 ^a,b,B^	0.37 ± 0.02 ^d,e,B^	1.20 ± 0.05 ^e,B^	3.89 ± 0.03 ^g,h,B^	5.51 ± 0.08 ^i,A^
**Ex-40.5**	0.05 ± 0.01 ^a,b,B^	0.41 ± 0.01 ^e,B^	1.21 ± 0.06 ^e,f,B^	3.74 ± 0.06 ^g,h,B^	5.41 ± 0.06 ^i,A^
**Ex-40.10**	0.16 ± 0.01 ^c,d,A^	0.35 ± 0.01 ^d,B^	1.27 ± 0.02 ^f,B^	4.33 ± 0.14 ^h,i,A^	6.11 ± 0.15 ^j,A^
**Commercial extruded rice**	0.12 ± 0.01 ^b,c^	0.30 ± 0.01 ^c^	0.75 ± 0.01 ^d^	1.01 ± 0.05 ^b^	2.25 ± 0.03 ^c^
***p* value**	<0.0001	<0.0001	<0.0001	<0.0001	<0.0001

* n.d. not detected. Values are mean ± standard error (*n* = 4); mean values in the same column followed by a different superscript are significantly (*p* < 0.05) different; small superscript letters mean differences between all the samples analysed, whereas capital superscript letters mean differences due to extrusion treatment for the same formulation. ** Sample codes: 20.0 (20% bean; 0% whole carob fruit); 20.5 (20% bean; 5% whole carob fruit); 20.10 (20% bean; 10% whole carob fruit); 40.0 (40% bean; 0% whole carob fruit); 40.5 (40% bean; 5% whole carob fruit); and 40.10 (40% bean; 10% whole carob fruit).

**Table 3:** Effect of extrusion treatment on trypsin inhibitors (TIU/mg dry weight), chymotrypsin inhibitors (CIU/mg dry weight), and lectin content (%PHA *) in raw materials, the non-extruded (NE-) and the extruded (Ex-) flour formulations, and the commercial sample.

Sample	Trypsin Inhibitors	Chymotrypsin Inhibitors	Lectins
**Bean**	23.21 ± 0.66 ^e^	7.74 ± 0.28 ^d^	0.297 ± 0.012 ^C^
**Carob fruit**	0.30 ± 0.02 ^a^	n.d. **	n.d.
**Rice**	0.15 ± 0.01 ^a^	n.d.	n.d.
NE-20.0 ***	4.10 ± 0.09 ^b,A^	1.97 ± 0.09 ^a,b,A^	0.035 ± 0.002 ^a^
**NE-20.5**	4.16 ± 0.06 ^b,A^	1.44 ± 0.08 ^a,A^	0.045 ± 0.002 ^a,b^
**NE-20.10**	5.53 ± 0.20 ^c,A^	1.66 ± 0.08 ^a,b,A^	0.052 ± 0.002 ^a,b^
**NE-40.0**	7.83 ± 0.11 ^d,B^	5.65 ± 0.17 ^c,B^	0.108 ± 0.005 ^a,b^
**NE-40.5**	7.73 ± 0.30 ^d,B^	5.93 ± 0.28 ^c,B^	0.103 ± 0.005 ^b^
**NE-40.10**	7.34 ± 0.29 ^d,B^	5.76 ± 0.25 ^c,B^	0.101 ± 0.005 ^a,b^
**Ex-20.0**	n.d.	n.d.	n.d.
**Ex-20.5**	n.d.	n.d.	n.d.
**Ex-20.10**	n.d.	n.d.	n.d.
**Ex-40.0**	n.d.	n.d.	n.d.
**Ex-40.5**	n.d.	n.d.	n.d.
**Ex-40.10**	n.d.	n.d.	n.d.
**Commercial extruded rice**	0.09 ± 0.01 ^a^	n.d.	n.d.
***p* value**	<0.0001	<0.0001	<0.0001

* PHA = *Phaseolus vulgaris* lectin. ** n.d. not detected. Values are mean ± standard error (*n* = 4); mean values in the same column followed by a different superscript are significantly (*p* < 0.05) different; small superscript letters mean differences between all the samples analysed, whereas capital superscript letters mean differences due to extrusion treatment for the same formulation. *** Sample codes: 20.0 (20% bean; 0% whole carob fruit); 20.5 (20% bean; 5% whole carob fruit); 20.10 (20% bean; 10% whole carob fruit); 40.0 (40% bean; 0% whole carob fruit); 40.5 (40% bean; 5% whole carob fruit); and 40.10 (40% bean; 10% whole carob fruit).

**Table 4:** Effect of extrusion on the anthocyanins (μg C3GlcE */g dry weight), flavonols (μg QE/g dry weight), tartaric esters (mg CAE/g dry weight), and total phenols (mg (+) CE/g dry weight) content and antioxidant activity (µmoles Trolox/g dry weight) of raw materials, the non-extruded (NE-) and the extruded (Ex-) flour formulations, and the commercial sample.

Sample	Anthocyanins	Flavonols	Tartaric Esters	Total Phenols	Antioxidant Activity (ORAC)
**Bean**	36.96 ± 0.24 ^i^	0.08 ± 0.001 ^c^	0.21 ± 0.01 ^i^	2.88 ± 0.02 ^g^	24.33 ± 0.07 ^i^
**Carob fruit**	18.00 ± 0.15 ^e^	0.75 ± 0.001 ^b,c^	0.72 ± 0.01 ^b,c^	20.73 ± 0.10 ^i^	69.89 ± 1.62 ^j^
**Rice**	18.70 ± 0.83 ^f^	0.03 ± 0.001 ^b^	0.02 ± 0.001 ^a^	0.90 ± 0.03 ^a,b^	3.80 ± 0.30 ^a^
NE-20.0 **	10.32 ± 0.07 ^a^	0.02 ± 0.001 ^a^	0.06 ± 0.001 ^b,A^	0.71 ± 0.03 ^a,A^	8.35 ± 0.04 ^b^
**NE-20.5**	11.00 ± 0.03 ^a,b,A^	0.04 ± 0.001 ^a,b,c^	0.09 ± 0.001 ^c,d,A^	1.38 ± 0.03 ^c, A^	9,66 ± 0.45 ^c,d,e^
**NE-20.10**	12.42 ± 0.23 ^a,A^	0.07 ± 0.001 ^b,c^	0.11 ± 0.001 ^e,f,A^	2.26 ± 0.01 ^e,f,A^	10.22 ± 0.50 ^d,e^
**NE-40.0**	15.23 ± 0.24 ^c^	0.03 ± 0.001 ^a,b,A^	0.09 ± 0.001 ^c,d,A^	1.10 ± 0.03 ^b,A^	9.41 ± 0.45 ^b,c,d^
**NE-40.5**	15.45 ± 0.36 ^c,d,A^	0.06 ± 0.001 ^a,b,c^	0.11 ± 0.001 ^e,f,A^	1.80 ± 0.03 ^d,A^	11.57 ± 0.50 ^f,g,h^
**NE-40.10**	17.08 ± 0.36 ^e,A^	0.08 ± 0.001 ^c^	0.14 ± 0.001 ^g,h,A^	2.35 ± 0.02 ^e,f,A^	12,36 ± 0.58 ^h,A^
**Ex-20.0**	12.41 ± 052 ^b^	0.02 ± 0.001 ^a^	0.07 ± 0.001 ^b,B^	0.92 ± 0.03 ^b,B^	8.92 ± 0.38 ^b,c^
**Ex-20.5**	16.78 ± 0.31 ^d,e,B^	0.05 ± 0.001 ^a,b,c^	0.10± 0.001 ^d,e,B^	2.16 ± 0.01 ^e,B^	9.32 ± 0.42 ^b,c,d^
**Ex-20.10**	17.13 ± 0.13 ^e,B^	0.08 ± 0.001 ^b,c^	0.14 ± 0.001 ^g,B^	3.25 ± 0.06 ^h,B^	10.29 ± 0.30 ^d,e^
**Ex-40.0**	18.13 ± 0.32 ^e^	0.08 ± 0.001 ^c,B^	0.08 ± 0.001 ^b,c,B^	1.33 ± 0.04 ^c,B^	10.49 ± 0.49 ^d,e,f^
**Ex-40.5**	21.19 ± 0.09 ^f,B^	0.06 ± 0.001 ^a,b,c^	0.13 ± 0.001 ^f,g,B^	2.37 ± 0.11 ^f,B^	11.89 ± 0.48 ^g,h^
**Ex-40.10**	23.30 ± 0.65 ^g,B^	0.08 ± 0.001 ^c^	0.16 ± 0.001 ^h,B^	3.17 ± 0.01 ^h,B^	10.86 ± 0.50 ^e,f,g,B^
**Commercial extruded rice**	15.59 ± 0.25 ^e,f^	0.06 ± 0.001 ^a,b,c^	0.14 ± 0.001 ^g^	1.96 ± 0.10 ^e^	8.81 ± 0.18 ^b,c^
***p* value**	<0.0001	<0.001	<0.0001	<0.0001	<0.0001

* C3GlcE (cyanidin-3-glucoside equivalents); QE (quercitin equivalents); CAE (caffeic acid equivalents); and CE ((+) catechin equivalents). Values are mean ± standard error (*n* = 4); mean values in the same column followed by a different superscript are significantly (*p* < 0.05) different; small superscript letters mean differences between all the samples analysed, whereas capital superscript letters mean differences due to extrusion treatment for the same formulation. ** Sample codes: 20.0 (20% bean; 0% whole carob fruit); 20.5 (20% bean; 5% whole carob fruit); 20.10 (20% bean; 10% whole carob fruit); 40.0 (40% bean; 0% whole carob fruit); 40.5 (40% bean; 5% whole carob fruit); and 40.10 (40% bean; 10% whole carob fruit).

## Supplementary Materials

The following are available online at https://www.mdpi.com/2304-8158/8/9/381/s1. Table S1. Values of the Principal Components (PC1, PC2, and PC3) coefficients for each formulation. Table S2. Component weight of each bioactive compound in relation to the Principal Components (PC1, PC2, and PC3). Figure S1. Principal component analysis (PCA) projection of the two first principal components. NE- (non-extruded formulations). Ex- (extruded formulations). Parameters: Total inositol phosphates (Total IP), sucrose, total galactosides, trypsin Inhibitors (TIU), chymotrypsin inhibitors (CIU), lectins (PHA), anthocyanins, flavonols, tartaric esters, total phenols, and ORAC. Figure S2. Principal component analysis (PCA) projection of the first and third principal components. NE- (non-extruded formulations). Ex- (extruded formulations). Parameters: Total inositol phosphates (Total IP), sucrose, total galactosides, trypsin Inhibitors (TIU), chymotrypsin inhibitors (CIU), lectins (PHA), anthocyanins, flavonols, tartaric esters, total phenols, and ORAC. Figure S3. Original HPLC Chromatograms of sucrose, galactinol, raffinose, ciceritol, and stachyose of the three raw materials, the six non-extruded (NE-), the six extruded (Ex-) flour formulations, and the extruded commercial sample. Figure S4. Original HPLC Chromatograms of inositol phosphates (IP3, IP4, IP5, and IP6) of the three raw samples, the six non-extruded (NE-), the six Extruded (EX-) flour formulations, and the extruded commercial sample.
