# Supplementary material for: Bioactive Compounds, Antioxidant Activity, and Sensory Analysis of Rice-Based Extruded Snacks-Like Fortified with Bean and Carob Fruit Flours
Source: Foods. 2019 Sep 2;8(9):381. doi: 10.3390/foods8090381 (PMC6770175; doi:10.3390/foods8090381)
Supplement: Supplementary file 1 [file foods-08-00381-s001.zip › Figure S4- HPLC Chromatograms- Inositol phosphates.pdf]

Bioactive compounds, antioxidant activity and sensory analysis of rice-based extruded snacks-like fortified with bean and carob fruit flours

Supplementary material

Figure S4. HPLC Chromatograms of inositol phosphates (IP3, IP4, IP5 and IP6) of the three raw samples, the six non-extruded (NE-), the six Extruded (EX-) flour formulations, and the extruded commercial sample

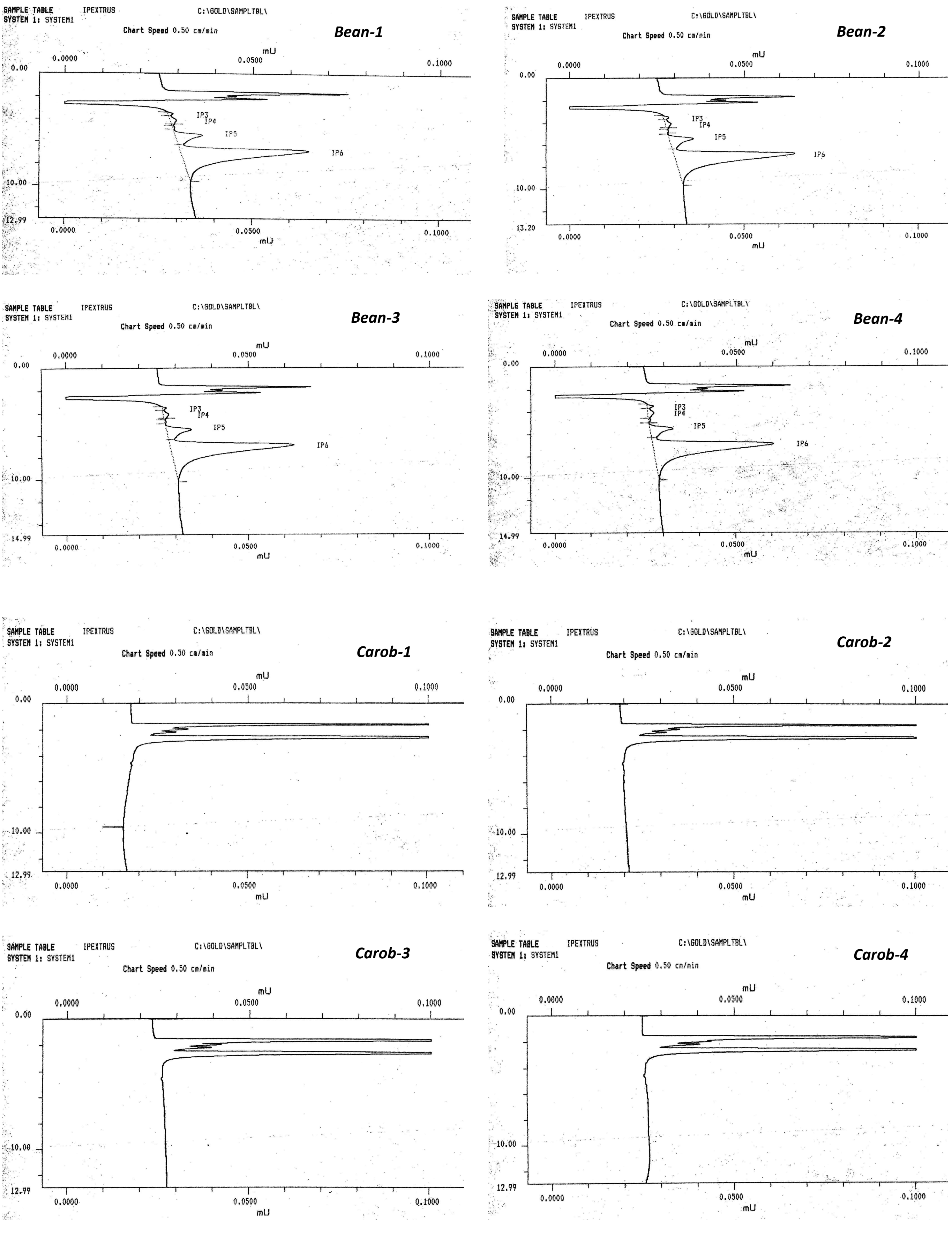

**Sample codes: 20.0 (20% bean; 0% whole carob fruit); 20.5 (20% bean; 5% whole carob fruit); 20.10 (20% bean; 10% whole carob fruit); 40.0 (40% bean; 0% whole carob fruit); 40.5 (40% bean; 5% whole carob fruit); 40.10 (40% bean; 10% whole carob fruit).

Rice-1

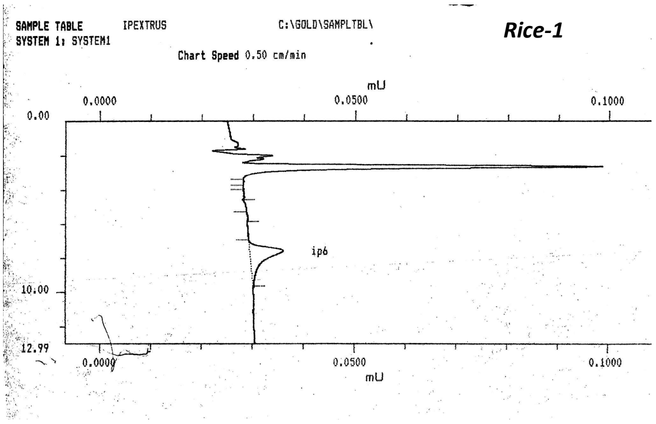

Rice-2

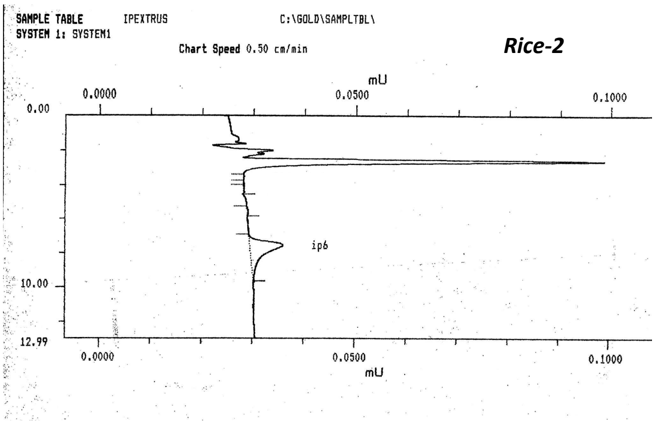

Rice-3

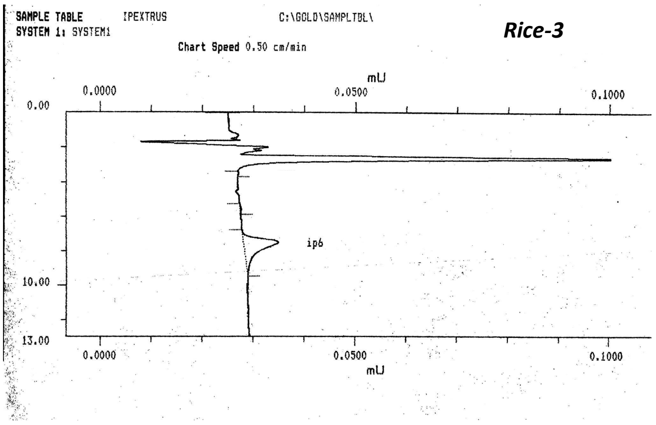

Rice-4

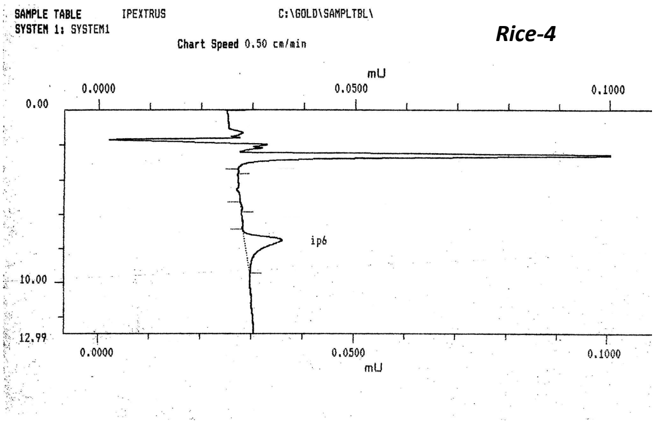

NE-20.0-1

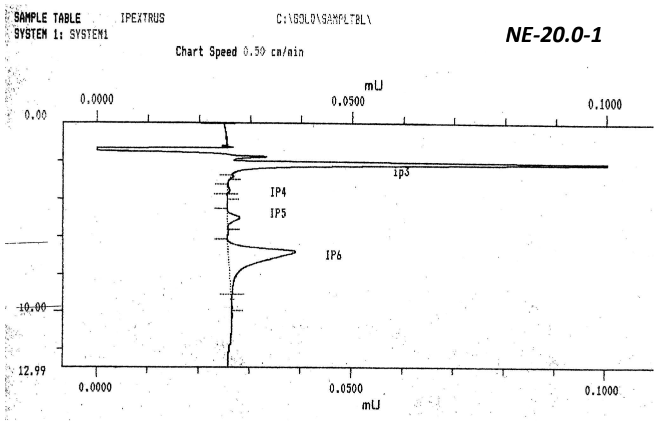

NE-20.0-2

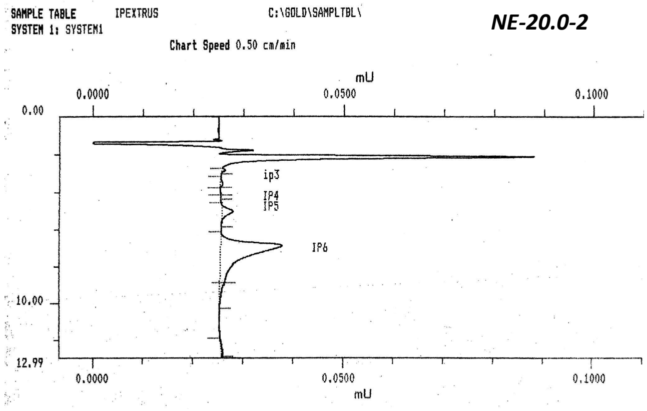

NE-20.0-3

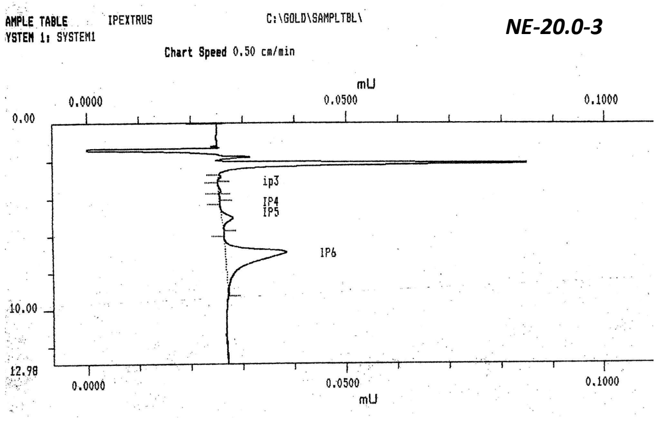

NE-20.0-4

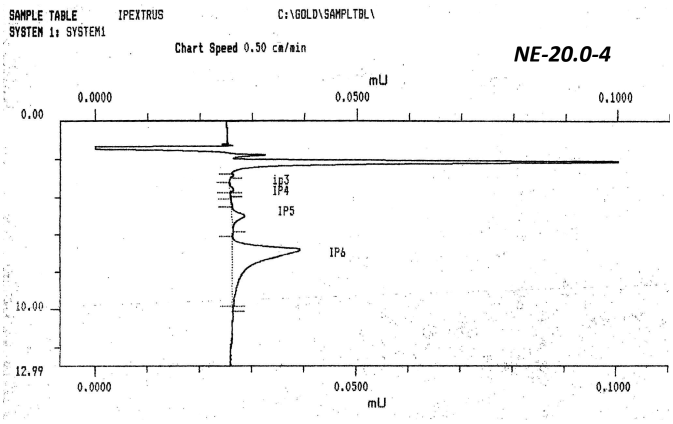

**Sample codes: 20.0 (20% bean; 0% whole carob fruit); 20.5 (20% bean; 5% whole carob fruit); 20.10 (20% bean; 10% whole carob fruit); 40.0 (40% bean; 0% whole carob fruit); 40.5 (40% bean; 5% whole carob fruit); 40.10 (40% bean; 10% whole carob fruit).

NE-20.5-1

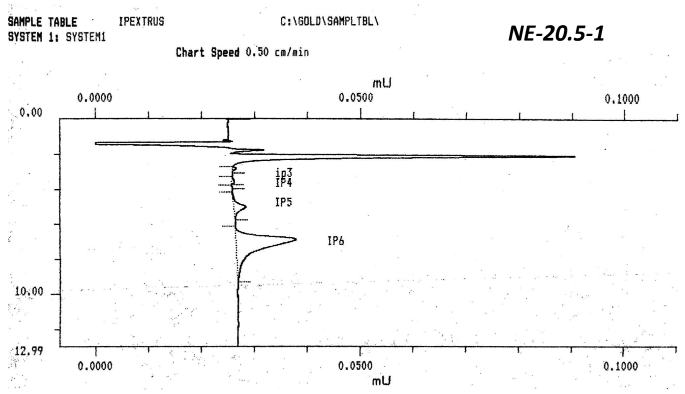

NE-20.5-2

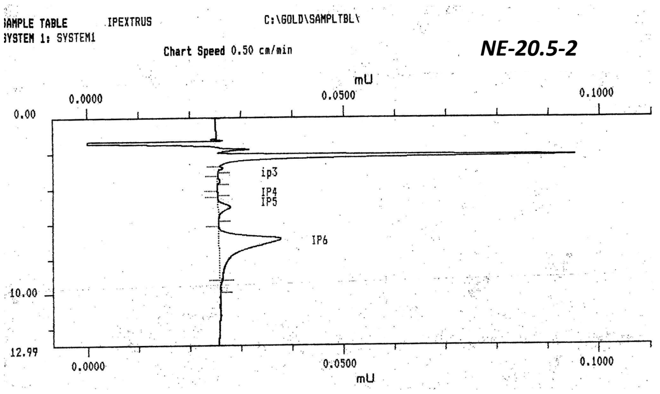

NE-20.5-3

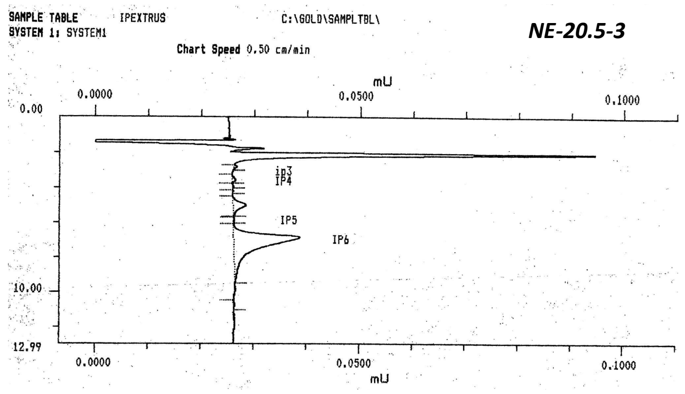

NE-20.5-4

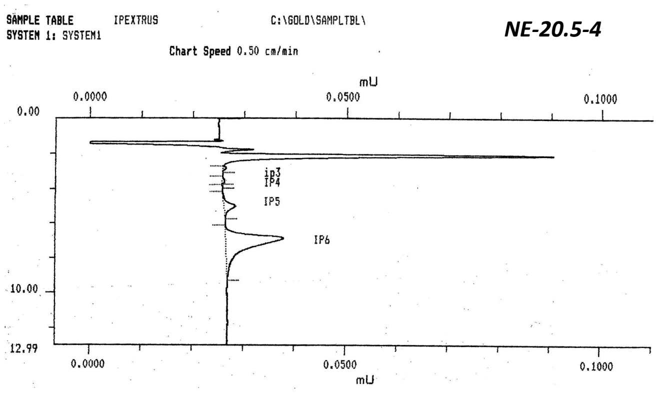

NE-20.10-1

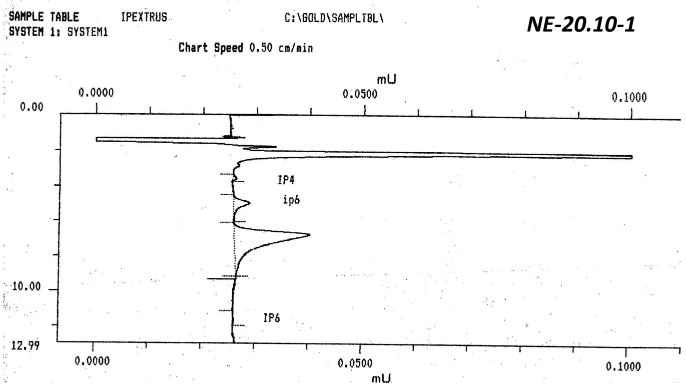

NE-20.10-2

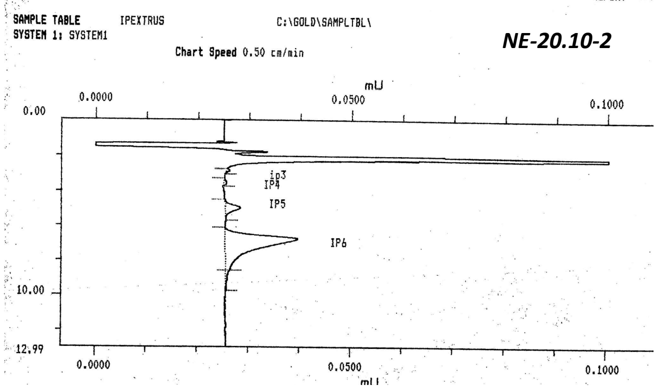

NE-20.10-3

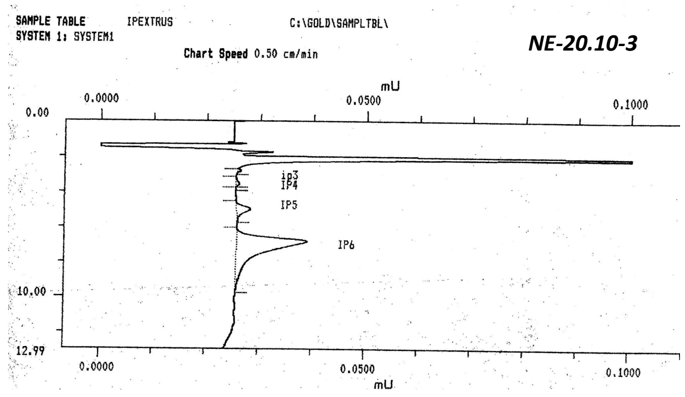

NE-20.10-4

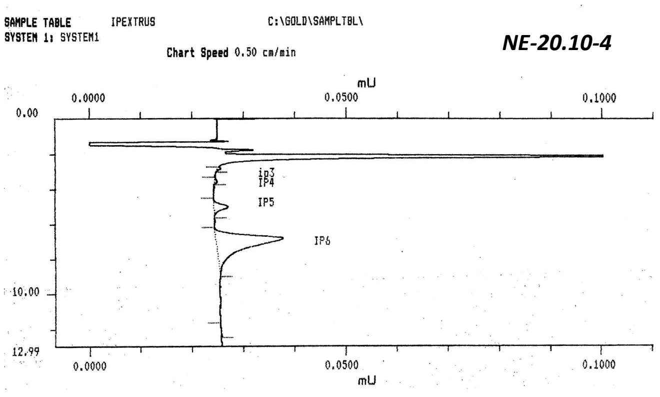

**Sample codes: 20.0 (20% bean; 0% whole carob fruit); 20.5 (20% bean; 5% whole carob fruit); 20.10 (20% bean; 10% whole carob fruit); 40.0 (40% bean; 0% whole carob fruit); 40.5 (40% bean; 5% whole carob fruit); 40.10 (40% bean; 10% whole carob fruit).

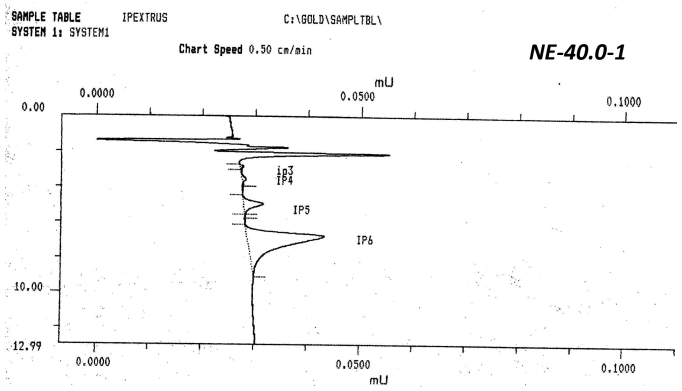

NE-40.0-1

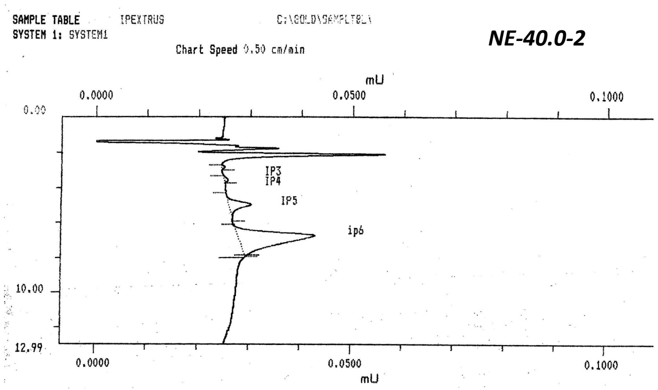

NE-40.0-2

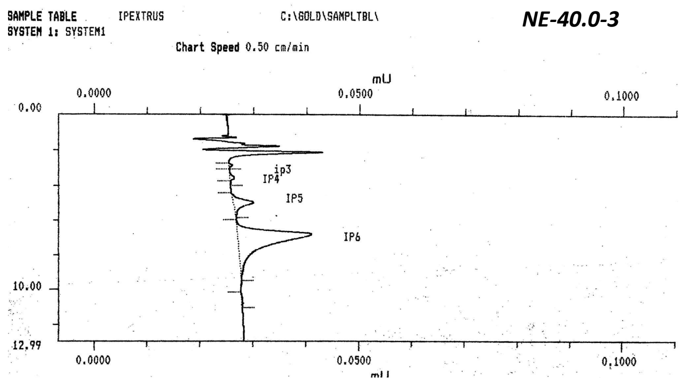

NE-40.0-3

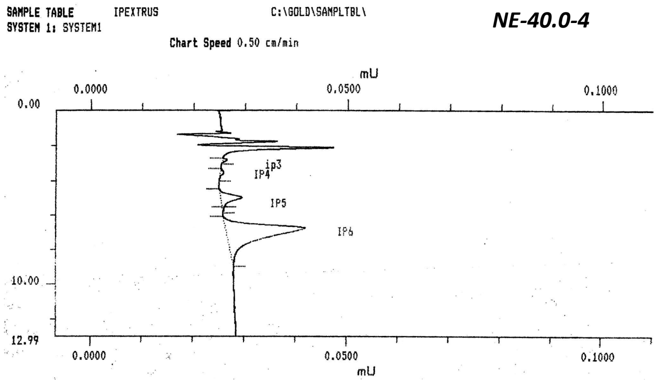

NE-40.0-4

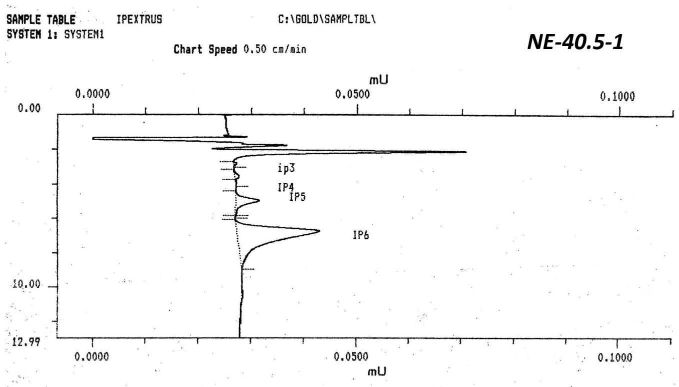

NE-40.5-1

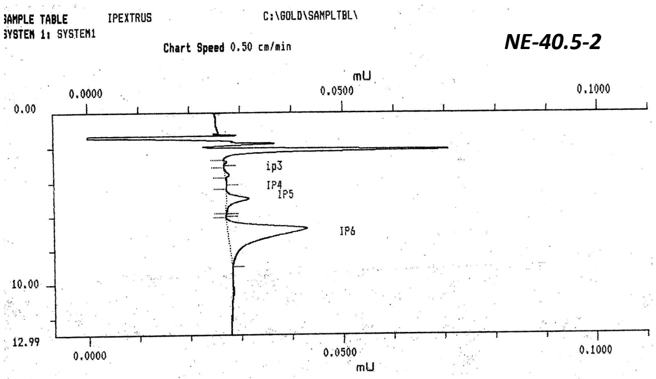

NE-40.5-2

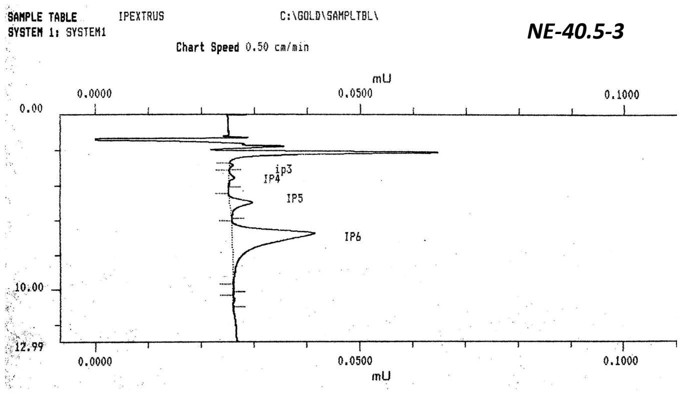

NE-40.5-3

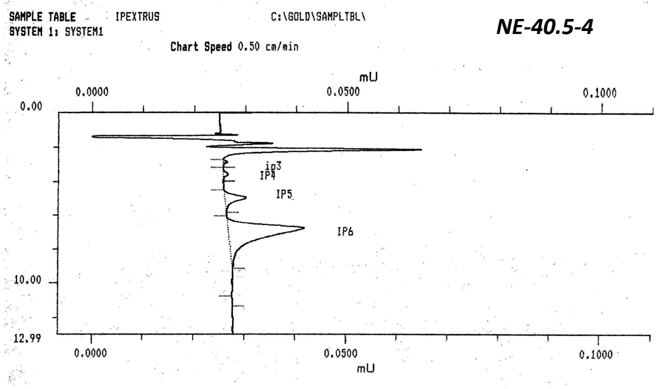

NE-40.5-4

**Sample codes: 20.0 (20% bean; 0% whole carob fruit); 20.5 (20% bean; 5% whole carob fruit); 20.10 (20% bean; 10% whole carob fruit); 40.0 (40% bean; 0% whole carob fruit); 40.5 (40% bean; 5% whole carob fruit); 40.10 (40% bean; 10% whole carob fruit).

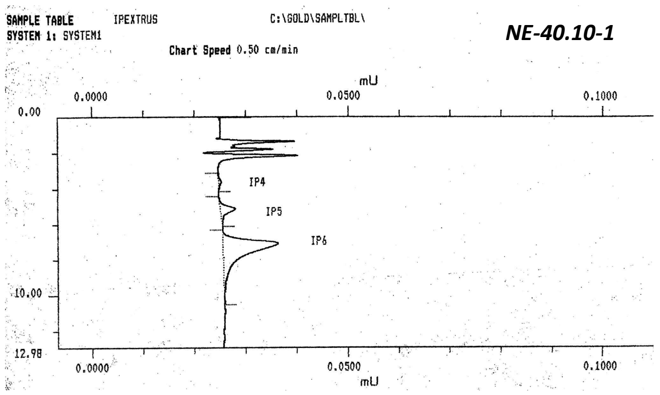

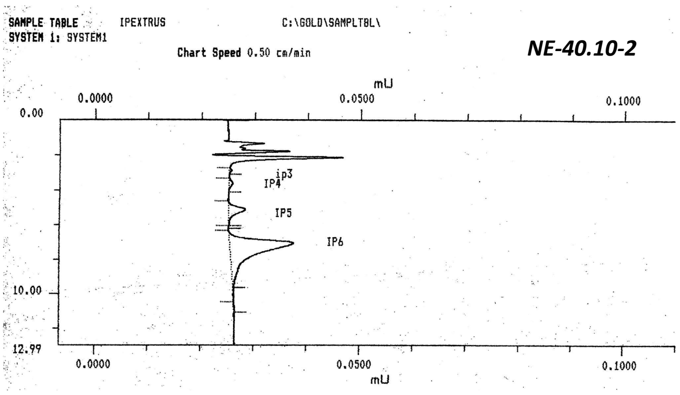

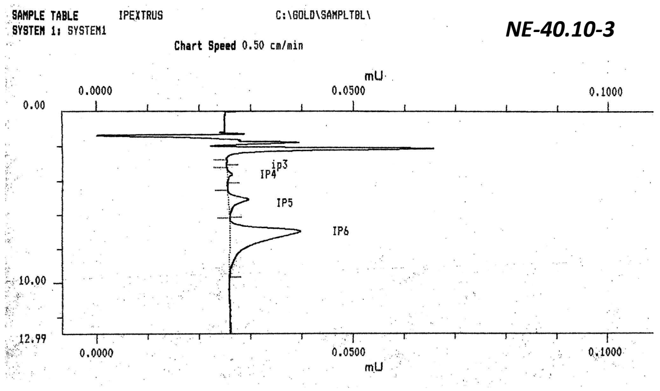

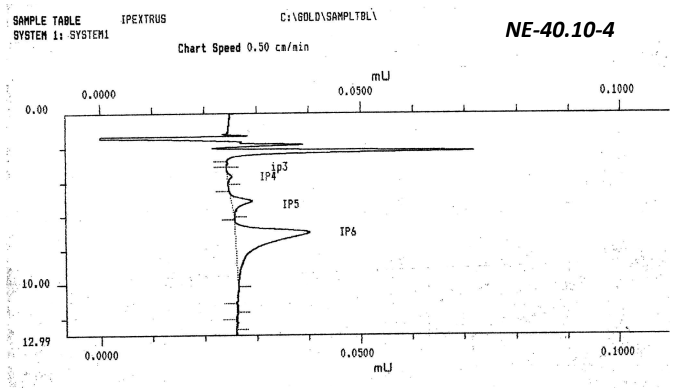

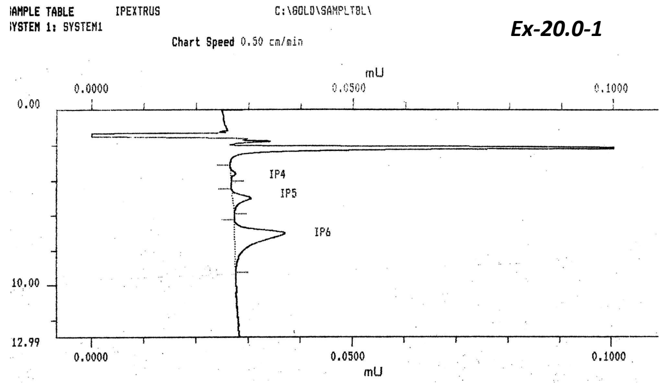

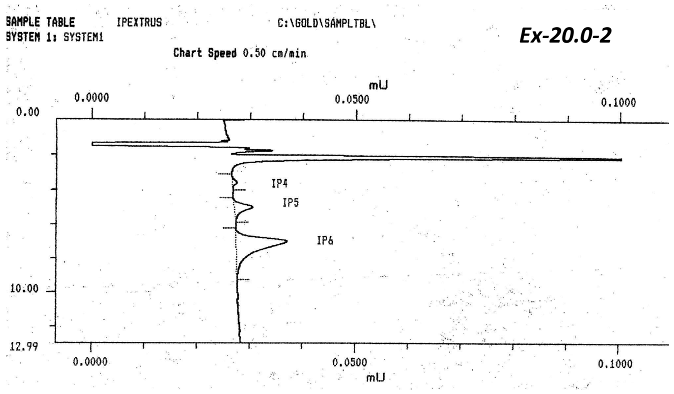

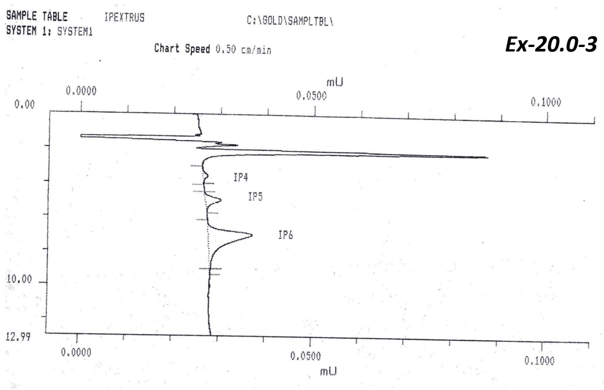

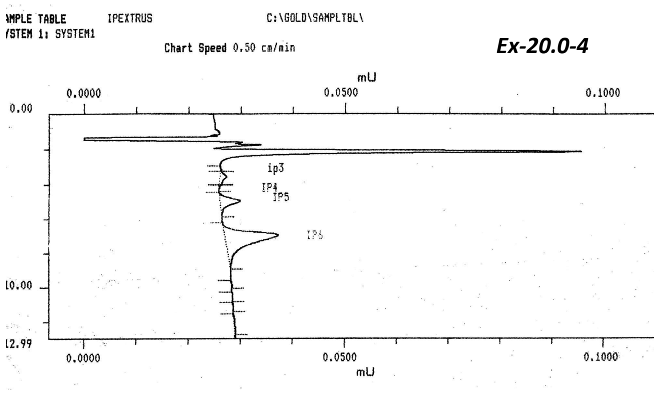

**Sample codes: 20.0 (20% bean; 0% whole carob fruit); 20.5 (20% bean; 5% whole carob fruit); 20.10 (20% bean; 10% whole carob fruit); 40.0 (40% bean; 0% whole carob fruit); 40.5 (40% bean; 5% whole carob fruit); 40.10 (40% bean; 10% whole carob fruit).

Ex-20.5-1

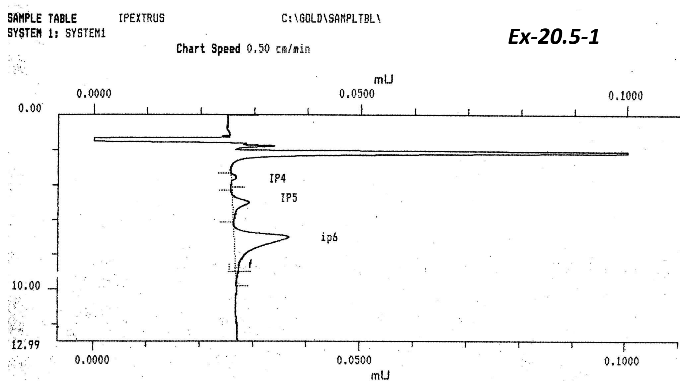

Ex-20.5-2

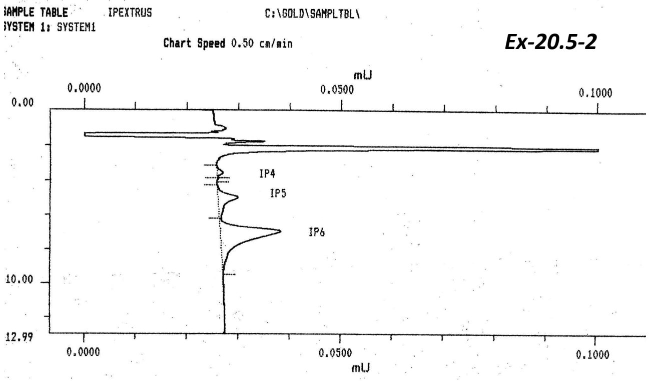

Ex-20.5-3

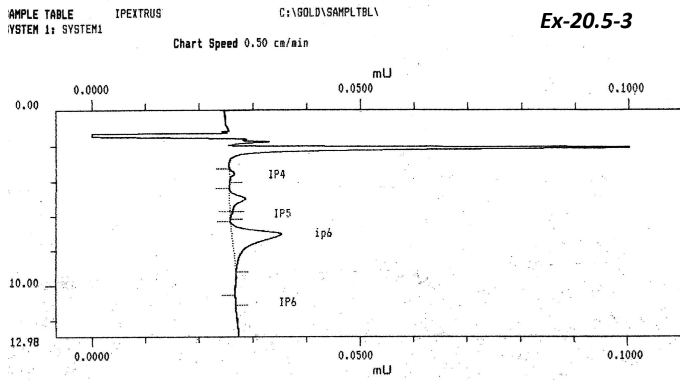

Ex-20.5-4

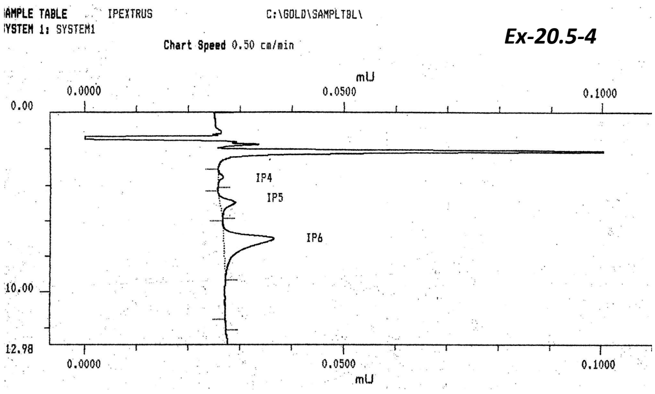

Ex-20.10-1

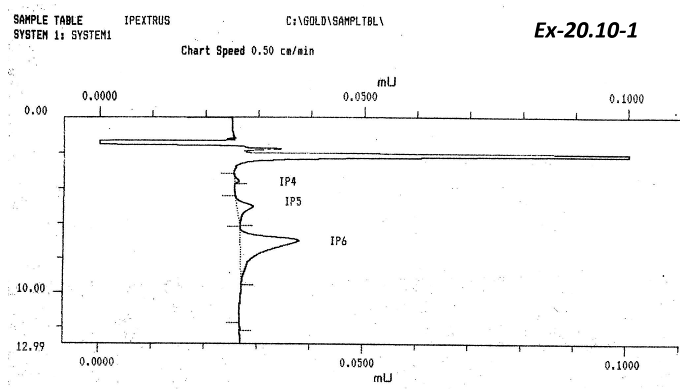

Ex-20.10-2

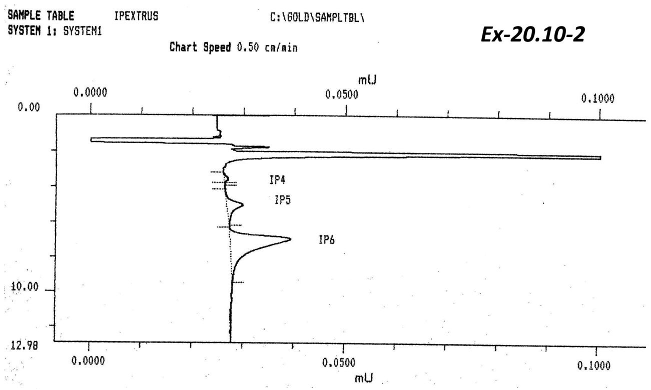

Ex-20.10-3

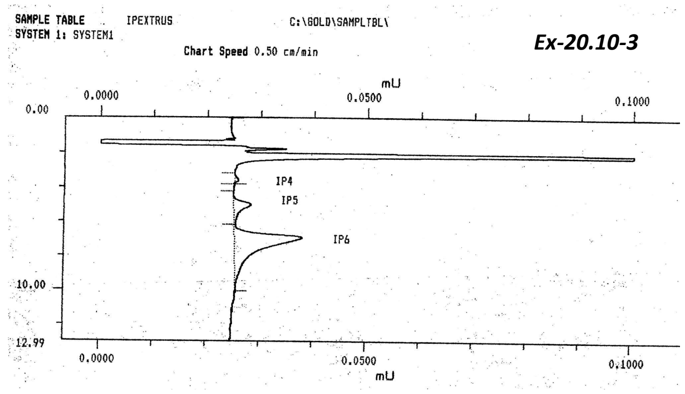

Ex-20.10-4

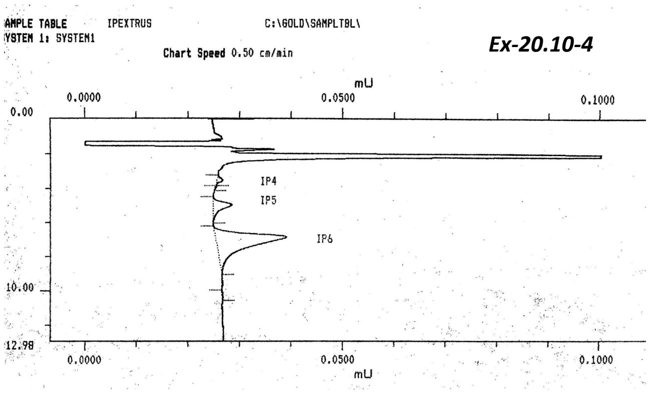

**Sample codes: 20.0 (20% bean; 0% whole carob fruit); 20.5 (20% bean; 5% whole carob fruit); 20.10 (20% bean; 10% whole carob fruit); 40.0 (40% bean; 0% whole carob fruit); 40.5 (40% bean; 5% whole carob fruit); 40.10 (40% bean; 10% whole carob fruit).

Ex-40.0-1

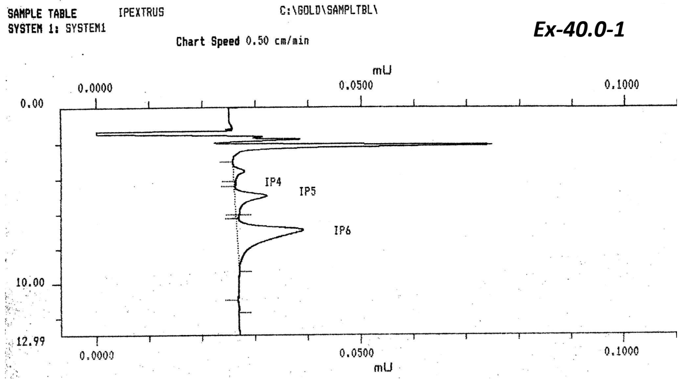

Ex-40.0-2

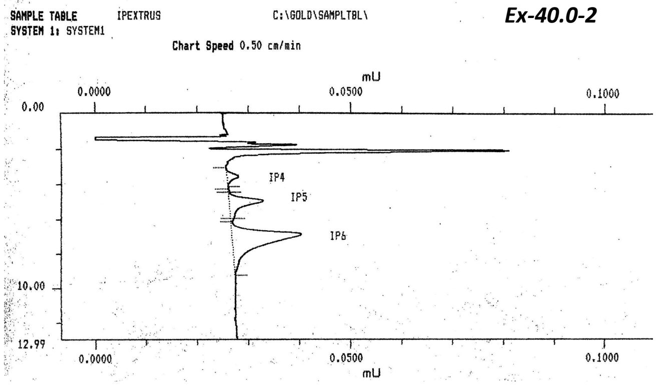

Ex-40.0-3

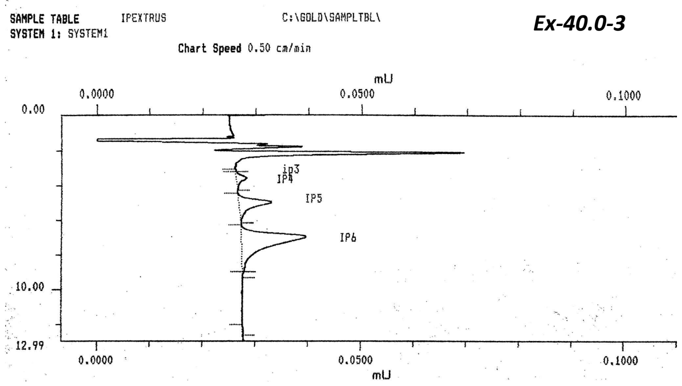

Ex-40.0-4

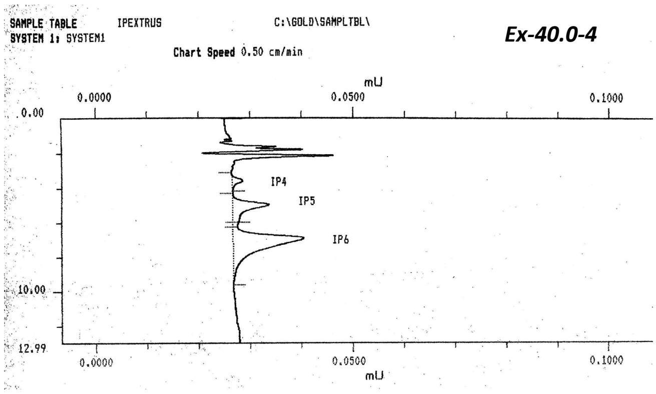

Ex-40.5-1

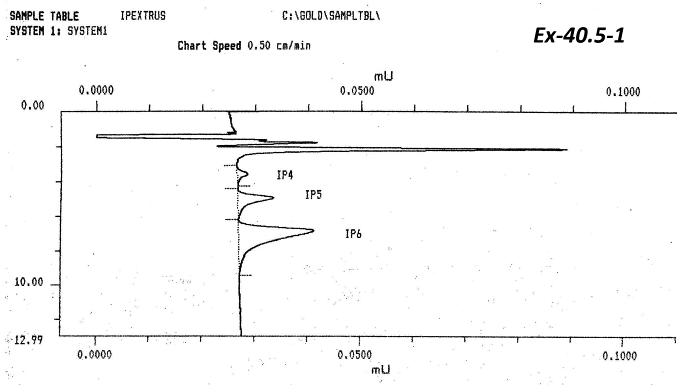

Ex-40.5-2

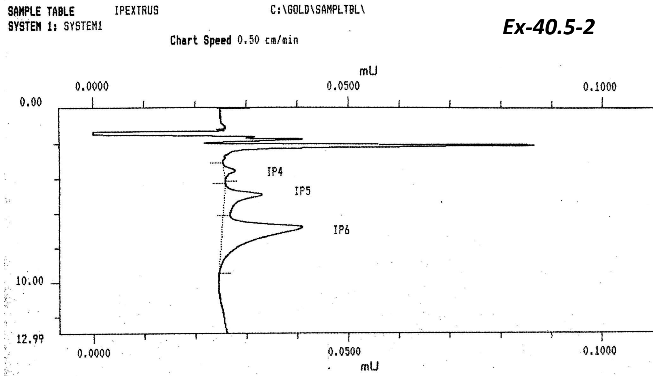

Ex-40.5-3

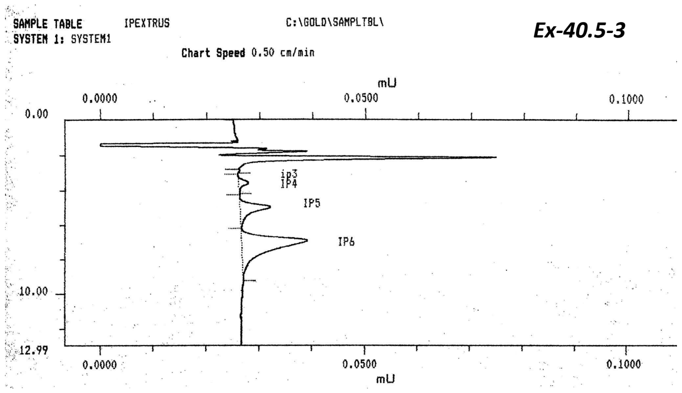

Ex-40.5-4

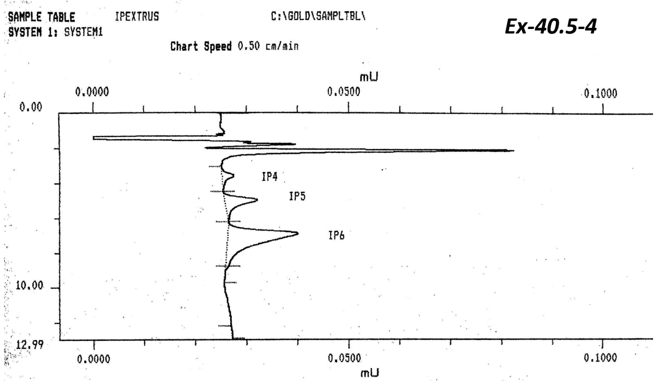

**Sample codes: 20.0 (20% bean; 0% whole carob fruit); 20.5 (20% bean; 5% whole carob fruit); 20.10 (20% bean; 10% whole carob fruit); 40.0 (40% bean; 0% whole carob fruit); 40.5 (40% bean; 5% whole carob fruit); 40.10 (40% bean; 10% whole carob fruit).

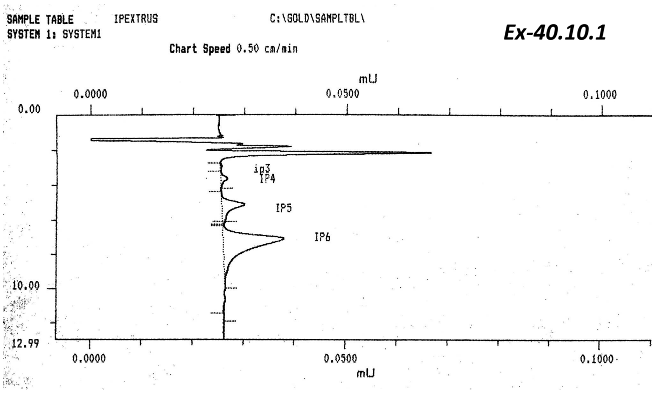

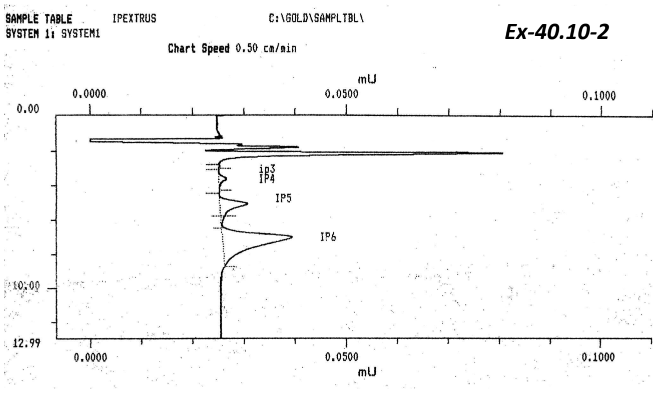

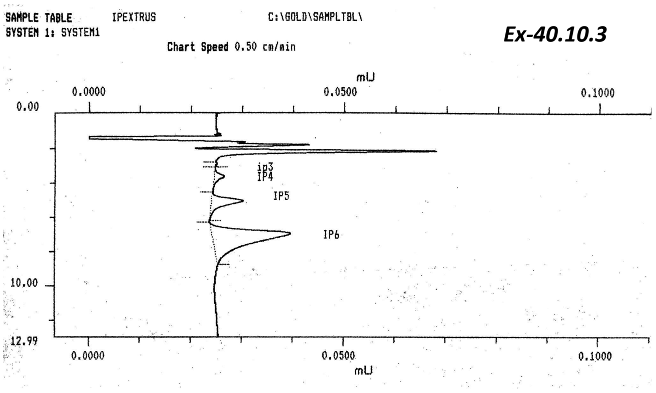

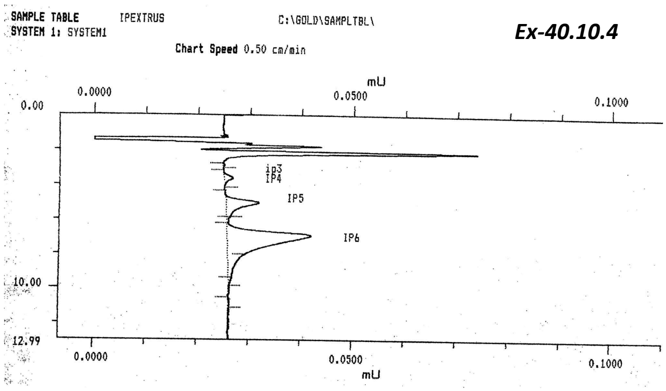

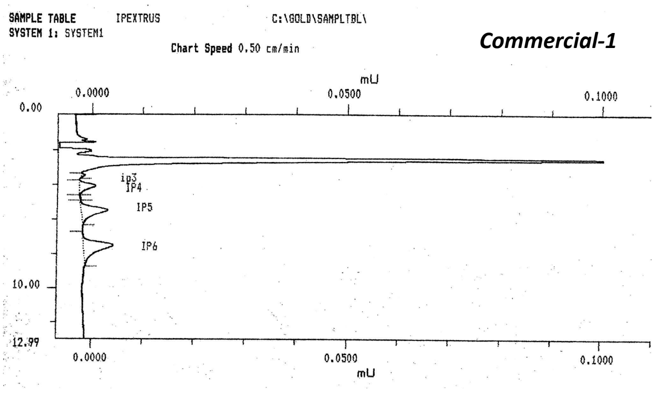

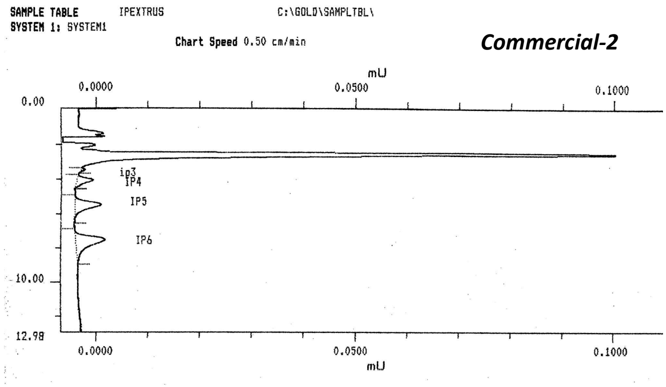

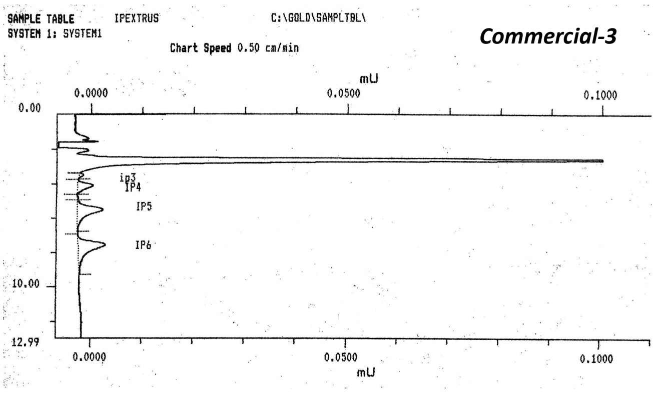

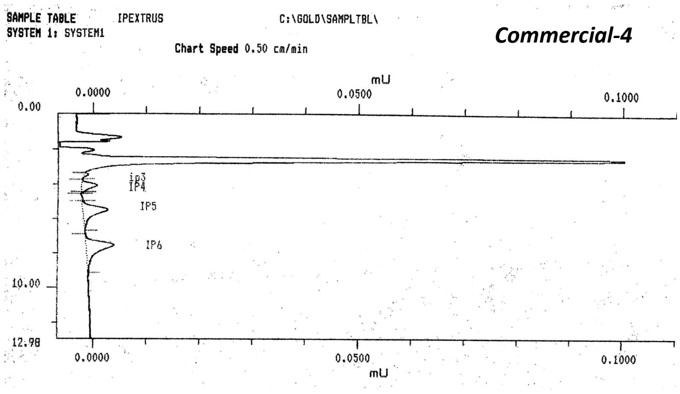

**Sample codes: 20.0 (20% bean; 0% whole carob fruit); 20.5 (20% bean; 5% whole carob fruit); 20.10 (20% bean; 10% whole carob fruit); 40.0 (40% bean; 0% whole carob fruit); 40.5 (40% bean; 5% whole carob fruit); 40.10 (40% bean; 10% whole carob fruit).
