# Supplementary material for: Bioactive Compounds, Antioxidant Activity, and Sensory Analysis of Rice-Based Extruded Snacks-Like Fortified with Bean and Carob Fruit Flours
Source: Foods. 2019 Sep 2;8(9):381. doi: 10.3390/foods8090381 (PMC6770175; doi:10.3390/foods8090381)

Bioactive compounds, antioxidant activity and sensory analysis of rice-based extruded snacks-like fortified with bean and carob fruit flours

Foods

SUPPLEMENTARY MATERIAL

Principal component analysis (PCA)

A multivariate analysis was performed to characterise and classify the different unprocessed (NE-) and processed (Ex-) rice-legume blends according to their bioactive compounds. The standardised data from the different bioactive compounds were subjected to a PCA to reduce the multidimensional structure of the data and to explain the observed variance that allows a meaningful interpretation of the measured variables and to ensure their reliability. Three principal components (PC) explained 86.85% of the total variance (47.91% for PC1, 29.61% for PC2 and 9.33% for PC3).

Table S1 shows the values of the three PCs for each formulation. PC1 and PC2 allowed characterising the samples concerning their treatment and legume percentage. In general, almost all the NE- formulations were negatively characterised by PC1 and positively characterised by PC2, while the Ex- formulations were positively characterised by PC1 and negatively characterised by PC2. The samples were classified in relation to the percentage of legume by PC2.

The weight of each measured bioactive compound for the three PCs is reported in Table S2. In general, the PC1 was weighted most heavily in a positive direction for the sucrose, the total galactosides and the different types of phenols analysed. The thermolabile compounds (trypsin and chymotrypsin inhibitors, lectins) and total inositol phosphates had more weight in the characterisation of the samples by PC2, while ORAC and flavonols had more weight in the characterisation of the samples by PC3.

All the compounds studied contributed to explain, to some extent, the variance of PC1, PC2, and PC3 (Fig. S1 and S2). Considering only PC1 and PC2, the NE blends were mainly characterised by both protease inhibitors and lectins, with the correlation sample/compounds was generally negative for PC1 and positive for PC2. Almost all the extruded samples were mainly positively characterised by total galactosides and anthocyanins in PC1 and negatively characterised in PC2.

From the PCA, it can be concluded that the extrusion process and the percentage of legume were well described by this analysis with the variance of PC1 explaining the treatment (extruded and non-extruded) and the variance of PC2 explaining the treatment and the percentage of legume.

Table S1. Values of the Principal Components (PC1, PC2, and PC3) coefficients for each formulation.

Formulation*	*PC 1*	*PC 2*	*PC3*
**NE-20.0**	-3.09772	-0.97039	0.53736
**NE-20.5**	-2.00266	0.08081	0.67893
**NE-20.10**	-0.34735	1.10810	-0.04889
**NE-40.0**	-2.45528	2.06767	-1.15031
**NE-40.5**	-0.29393	2.17311	0.43487
**NE-40.10**	0.75376	3.09486	-0.13308
**Ex-20.0**	-2.5238	-2.78136	-0.38727
**Ex-20.5**	-0.01258	-1.41516	1.38064
**Ex-20.10**	2.22062	-0.50673	0.56503
**Ex-40.0**	1.06972	-1.73968	-2.43473
**Ex-40.5**	2.45624	-0.88705	0.87740
**Ex-40.10**	4.23297	-0.22418	-0.31994

*Sample codes: 20.0 (20% bean; 0% whole carob fruit); 20.5 (20% bean; 5% whole carob fruit); 20.10 (20% bean; 10% whole carob fruit); 40.0 (40% bean; 0% whole carob fruit); 40.5 (40% bean; 5% whole carob fruit); 40.10 (40% bean; 10% whole carob fruit).

Table S2. Component weight of each bioactive compound in relation to the Principal Components (PC1, PC2, and PC3).

Bioactive Compound	*PC 1*	*PC 2*	*PC3*
**Total Inositol phosphates**	0.31427	0.33558	-0.29188
**Sucrose**	0.40407	0.12776	0.19838
**Total galactosides**	0.32443	-0.23484	-0.31506
**TIU**	-0.19347	0.49989	-0.04273
**CIU**	-0.12844	0.49686	-0.10172
**Anthocyanins**	0.39562	-0.05338	-0.14111
**Flavonols**	0.30489	0.04481	-0.50255
**Tartaric esters**	0.35341	0.27322	0.16141
**Total Phenols**	0.37672	0.11176	0.28381
**ORAC**	0.17042	0.17367	0.58488
**PHA**	-0.18289	0.44384	-0.20702

Figure S1. Principal component analysis (PCA) projection of the two first principal components. NE- (non-extruded formulations). Ex- (extruded formulations). Parameters: total inositol phosphates (Total IP), sucrose, total galactosides, trypsin Inhibitors (TIU), chymotrypsin inhibitors (CIU), lectins (PHA), anthocyanins, flavonols, tartaric esters, total phenols, and ORAC.

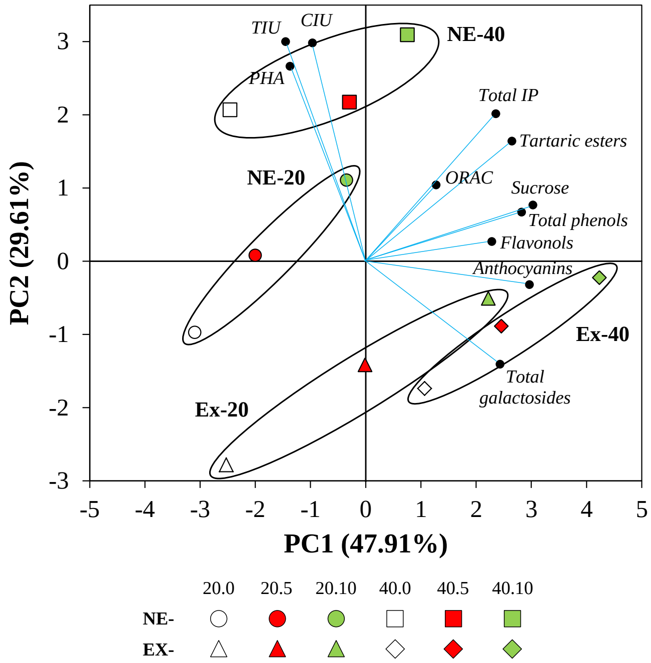

*Sample codes: 20.0 (20% bean; 0% whole carob fruit); 20.5 (20% bean; 5% whole carob fruit); 20.10 (20% bean; 10% whole carob fruit); 40.0 (40% bean; 0% whole carob fruit); 40.5 (40% bean; 5% whole carob fruit); 40.10 (40% bean; 10% whole carob fruit).

Figure S2. Principal component analysis (PCA) projection of the first and third principal components. NE- (non-extruded formulations). Ex- (extruded formulations). Parameters: total inositol phosphates (Total IP), sucrose, total galactosides, trypsin Inhibitors (TIU), chymotrypsin inhibitors (CIU), lectins (PHA), anthocyanins, flavonols, tartaric esters, total phenols, and ORAC.

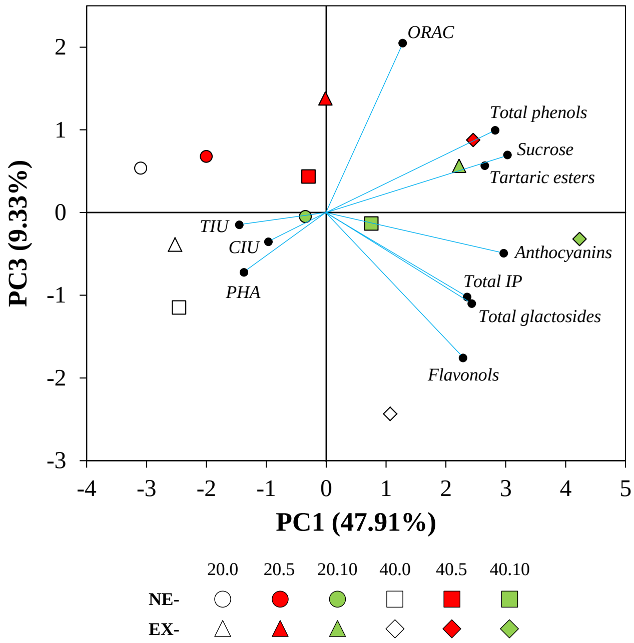

Supplement: Supplementary file 1 [file foods-08-00381-s001.zip › Supplementary Principal Components Analysis.pdf]
